# RSPO3 is important for trabecular bone and fracture risk in mice and humans

**DOI:** 10.1038/s41467-021-25124-2

**Published:** 2021-08-13

**Authors:** Karin H. Nilsson, Petra Henning, Maha El Shahawy, Maria Nethander, Thomas Levin Andersen, Charlotte Ejersted, Jianyao Wu, Karin L. Gustafsson, Antti Koskela, Juha Tuukkanen, Pedro P. C. Souza, Jan Tuckermann, Mattias Lorentzon, Linda Engström Ruud, Terho Lehtimäki, Jon H. Tobias, Sirui Zhou, Ulf H. Lerner, J. Brent Richards, Sofia Movérare-Skrtic, Claes Ohlsson

**Affiliations:** 1 Sahlgrenska Osteoporosis Centre, Centre for Bone and Arthritis Research, Institute of Medicine, Sahlgrenska Academy at University of Gothenburg, Gothenburg, Sweden; 2 Department of Clinical Research, University of Southern Denmark, Odense, Denmark; 3 Department of Pathology, Odense University Hospital, Odense, Denmark; 4 Department of Endocrinology, Odense University Hospital, Odense, Denmark; 5 Department of Anatomy and Cell Biology, Faculty of Medicine, Institute of Cancer Research and Translational Medicine, University of Oulu, Oulu, Finland; 6 Innovation in Biomaterials Laboratory, Faculty of Dentistry, Federal University of Goiás, Goiâna, Brazil; 7 Institute of Comparative Molecular Endocrinology (CME), University of Ulm, Ulm, Germany; 8 Region Västra Götaland, Department of Geriatric Medicine, Sahlgrenska University Hospital, Mölndal, Sweden; 9 Mary MacKillop Institute for Health Research, Australian Catholic University, Melbourne, VIC Australia; 10 Department of Physiology, Institute of Neuroscience and Physiology, Sahlgrenska Academy at the University of Gothenburg, Gothenburg, Sweden; 11 Department of Clinical Chemistry, Fimlab Laboratories, Tampere, Finland; 12 Finnish Cardiovascular Research Center – Tampere, Faculty of Medicine and Health Technology, Tampere University, Tampere, Finland; 13 Musculoskeletal Research Unit, Translational Health Sciences, and Medical Research Council Integrative Epidemiology Unit, Bristol Medical School, University of Bristol, Bristol, UK; 14 Department of Medicine, Centre for Clinical Epidemiology, Lady Davis Institute, Jewish General Hospital, McGill University, Montréal, QC Canada; 15 Department of Human Genetics, McGill University, Montréal, QC Canada; 16 Region Västra Götaland, Department of Drug Treatment, Sahlgrenska University Hospital, Gothenburg, Sweden; 17 Present Address: Faculty of Dentistry, Department of Oral Biology, Minia University, Minia, Egypt

**Keywords:** Osteoblasts, Bone

## Abstract

With increasing age of the population, countries across the globe are facing a substantial increase in osteoporotic fractures. Genetic association signals for fractures have been reported at the *RSPO3* locus, but the causal gene and the underlying mechanism are unknown. Here we show that the fracture reducing allele at the *RSPO3* locus associate with increased RSPO3 expression both at the mRNA and protein levels, increased trabecular bone mineral density and reduced risk mainly of distal forearm fractures in humans. We also demonstrate that RSPO3 is expressed in osteoprogenitor cells and osteoblasts and that osteoblast-derived RSPO3 is the principal source of RSPO3 in bone and an important regulator of vertebral trabecular bone mass and bone strength in adult mice. Mechanistic studies revealed that RSPO3 in a cell-autonomous manner increases osteoblast proliferation and differentiation. In conclusion, RSPO3 regulates vertebral trabecular bone mass and bone strength in mice and fracture risk in humans.

## Abstract (web summary)

Genetic association signals for fractures have been reported at the *RSPO3* locus, but the causal gene and the underlying mechanism are unknown. Here, the authors show that RSPO3 exerts an important role for vertebral trabecular bone mass and bone strength in mice and fracture risk in humans.

## Introduction

Osteoporosis is a common skeletal disease, leading to a reduction in bone density and quality, and an increased fracture risk. One in two elderly women and one in four elderly men will at some point suffer from an osteoporotic fracture^1,2^. It is predicted that the number of people aged 65 years and older will triple globally by year 2100^3,4^ as compared to those aged 12–64 years, and the incidence of fractures increases exponentially with age^5^. Therefore, the identification and treatment of patients at high fracture risk is an important public health goal.

Fracture risk is a moderately heritable trait^6,7^, and two recent large-scale genome-wide association studies (GWAS) have identified 15 loci associated with fractures at any bone site^3,8^. The identified fracture loci were all also associated with BMD, supporting the notion that BMD is a major regulator of fracture risk^3,8^. These previous large-scale human genetic studies reported that the strongest genetic determinant for fractures is located at the *WNT16* locus and detailed translational studies revealed that osteoblast-derived WNT16 increases cortical bone mass^9–11^. The second strongest genetic determinant for fractures at any bone site (*P* = 8.9 × 10^−65^)^8^ is located at the *RSPO3* locus. RSPO3 is a known WNT-signaling modulator^12,13^, and WNT-signaling is a major determinant of bone density and strength. We therefore hypothesized in this study that RSPO3 is the causal gene for the prominent fracture signal at the *RSPO3* locus.

WNT proteins belong to a family of secreted cysteine-rich glycoproteins that signal through both the WNT–β-catenin pathway, also termed the canonical WNT pathway, and noncanonical WNT pathways^13–16^. WNT ligands increase bone mass by targeting the trabecular and/or cortical bone compartments^10,17^. WNT16 is the major known cortical bone-specific WNT^10^, while WNT10b protects against age-dependent trabecular bone loss^18,19^. WNT binding to its Frizzled receptors leads to nuclear accumulation of the transcriptional co-activator β-catenin and, thereby, increased WNT target gene expression^12^. There are a number of secreted extracellular proteins including sclerostin, DKK1 and NOTUM that inhibit WNT-signaling and thereby reduce bone mass^12,20^. Another group of secreted proteins, R-spondins (RSPO 1–4), are described to amplify WNT-signaling^21–26^. They are ligands of the leucine-rich repeat-containing G-protein coupled receptors (LGRs), including LGR4, -5, and -6^27–29^, and reduce degradation of Frizzled receptors and consequently increase WNT-signaling^23,28,30,31^. The in vivo role of RSPO3 for adult bone metabolism is not known as global *Rspo3*^−/−^ mice display early embryonic lethality due to impaired formation of the placenta^29^.

Previous human genetic studies have reported that the fracture reducing C-allele of rs7741021, the strongest genetic determinant for fractures at the *RSPO3* locus, also is robustly associated with increased BMD (Table 1a)^8^. However, the causal gene(s) and the underlying mechanism are unknown. In addition, it is unknown if its associations with fractures at different bone sites differ and if the association with BMD is mediated via trabecular and/or cortical bone parameters. We, herein, used a combination of human genetic studies and extensive functional mechanistic studies to identify the causal gene at the *RSPO3* locus and its mechanism to explain its effect upon fracture.

**Table 1:** (a) Associations for rs7741021 in the *RSPO3* locus (C-allele = effect allele). (b) Associations for rs3734626 in the *RSPO3* locus (T-allele = effect allele). (c) Associations for rs2489623 in the *RSPO3* locus (C-allele = effect allele).

Trait	EAF	OR	95% CI	*P* -value	No. of cases	No. of controls	Source
(a)							
***Fractures***							
Fracture at any bone site	0.47	0.95	(0.94–0.96)	8.9E−65	421,084	737,530	Morris et al. (Table S4)^8^
Distal forearm fractures	0.48	0.88	(0.86–0.91)	9.6E−14	7,324	431,432	New analyses in UK Biobank
Hip fractures	0.48	0.94	(0.90–0.98)	4.4E−03	4,035	434,902	New analyses in UK Biobank
***Bone parameters***	**EAF**	**Beta**	**SE**	***P*-value**	**No. of total**		**Source**
eBMD	0.48	0.079	0.002	5.6E−336	426,824		Morris et al. ^8^
Trabecular vBMD	0.48	0.088	0.028	1.4E−03	2,500		Paternoster et al. ^34^
Cortical vBMD	0.48	−0.013	0.015	4.1E−01	5,878		Paternoster et al. ^34^
Cortical thickness	0.48	0.012	0.017	4.8E−01	5,878		Zheng et al. ^9^
***eQTL***	**EAF**	**Beta**	**SE**	***P*value**	**No. of total**		**Source**
RSPO3 mRNA in adipose tissue	0.44	0.15*		2.4E−04	581		GTEx portal
RSPO3 mRNA in fibroblasts	0.46	0.12*		1.8E−04	483		GTEx portal

(b)							
***Fractures***							
Fracture at any bone site	0.53	0.94	(0.93–0.95)	1.8E−20	53,184	426,795	Morris et al. ^8^
Distal forearm fractures	0.52	0.89	(0.86–0.92)	2.4E−12	7,324	431,432	New analyses in UK Biobank
Hip fractures	0.52	0.95	(0.91–0.99)	1.4E−02	4,035	434,902	New analyses in UK Biobank
***Bone parameters***	**EAF**	**Beta**	**SE**	***P*value**	**No. of total**		**Source**
eBMD	0.53	0.069	0.002	1.9E−258	426,824		Morris et al. ^8^
Trabecular vBMD	0.51	0.092	0.028	9.4E−04	2,500		Paternoster et al. ^34^
Cortical vBMD	0.52	−0.010	0.015	5.0E−01	5,878		Paternoster et al. ^34^
Cortical thickness	0.52	0.015	0.017	3.7E−01	5,878		Zheng et al. ^9^
***cis-pQTL***	**EAF**	**Beta**	**SE**	***P*value**	**No. of total**		**Source**
Circulating RSPO3	0.56	0.325	0.023	1.8E−45	3,200		Emilsson et al. (Table S13)^33^

(c)							
**Fractures**							
Fracture at any bone site	0.54	0.95	(0.94–0.96)	1.0E−14	53,184	426,795	Morris et al. ^8^
Distal forearm fractures	0.53	0.89	(0.86–0.92)	8.5E–13	7,324	431,432	New analyses in UK Biobank
Hip fractures	0.53	0.96	(0.92–1.01)	1.1E–01	4,035	434,902	New analyses in UK Biobank
**Bone parameters**	**EAF**	**Beta**	**SE**	***P*value**	**No. of total**		**Source**
eBMD	0.54	0.061	0.002	6.6E−205	426,824		Morris et al. ^8^
Trabecular vBMD	0.53	0.084	0.028	2.4E−03	2,500		Paternoster et al. ^34^
Cortical vBMD	0.53	0.020	0.015	2.0E−01	5,878		Paternoster et al. ^34^
Cortical thickness	0.53	0.016	0.017	3.5E−01	5,878		Zheng et al. ^9^
**cis-pQTL**	**EAF**	**Beta**	**SE**	***P*value**	**No. of total**		**Source**
RSPO3	0.53	0.270	0.025	3.6E−28	3,301		Sun et al, (Table S4)^35^

(a) Betas are given in SD (or ORs) per effect allele (=C-allele). *The effect sizes for the eQTLs are given as normalized effect size according to the GTEx portal (https://gtexportal.org/home). eBMD = estimated bone mineral density measured by ultrasound in the heel. Trabecular vBMD = Trabecular volumetric BMD as measured by pQCT in the distal tibia metaphysis. Cortical vBMD = Cortical volumetric BMD as measured by pQCT in the tibia diaphysis. Cortical thickness = Cortical thickness measured by pQCT in the diaphysis of tibia. eQTL = expression quantitative trait locus. The effect size for the association of rs7741021 with forearm fractures was significantly higher than the effect size for the association of rs7741021 with fractures at any bone site (*P* = 1.6 × 10^−5^; *Z*-test) and hip fractures (*P* = 3.5 × 10^−2^; *Z*-test). The statistical tests were two-sided.

(b) Betas are given in SD (or ORs) per effect allele (=T-allele). eBMD = estimated bone mineral density measured by ultrasound in the heel. Trabecular vBMD = Trabecular volumetric BMD as measured by pQCT in the distal tibia metaphysis. Cortical vBMD = Cortical volumetric BMD as measured by pQCT in the tibia diaphysis. Cortical thickness = Cortical thickness measured by pQCT in the diaphysis of tibia. pQTL = protein quantitative trait locus. The effect size for the association of rs3734626 with forearm fractures was significantly higher than the effect size for the association of rs3734626 with fractures at any bone site (*P* = 2.1 × 10^−3^; *Z*-test) and hip fractures (*P* = 3.0 × 10^−2^; *Z*-test). The statistical tests were two-sided.

(c) Betas are given in SD (or ORs) per effect allele (=C-allele). eBMD = estimated bone mineral density measured by ultrasound in the heel. Trabecular vBMD = Trabecular volumetric BMD as measured by pQCT in the distal tibia metaphysis. Cortical vBMD = Cortical volumetric BMD as measured by pQCT in the tibia diaphysis. Cortical thickness = Cortical thickness measured by pQCT in the diaphysis of tibia. pQTL = protein quantitative trait locus. The effect size for the association of rs2489623 with forearm fractures was significantly higher than the effect size for the association of rs2489623 with fractures at any bone site (*P* = 2.1 × 10^−3^; *Z*-test) and hip fractures (*P* = 3.0 × 10^−2^; *Z*-test). The statistical tests were two-sided.

## Results

### Human genetic studies indicate that increased RSPO3 expression is associated with increased trabecular bone mineral density and reduced risk of distal forearm fractures

Previous large-scale human genetic studies have reported that the strongest genetic determinant at the *RSPO3* locus for fractures at any bone site is located at the intronic SNP rs7741021 (Table 1a; *P* = 8.9 × 10^−65^)^8^. A substantial part of the SNPs associated with evaluated phenotypes in GWAS is located in intronic regions and may directly or via highly linked SNPs affect regulatory regions and thereby gene transcription^32^. We, therefore, evaluated the association between rs7741021 and mRNA levels of RSPO3 using the GTEx resource. We observed that the C-allele of rs7741021, previously reported to associate with reduced fracture risk at any bone site and increased BMD (estimated BMD as determinated by ultrasound in the heel; Table 1a), was associated with increased *RSPO3* mRNA levels in both subcutaneous adipose tissue (*P* = 2.4 × 10^−4^; *n* = 581) and cultured fibroblasts (*P* = 1.8 × 10^−4^; *n* = 483; Table 1a). Unfortunately, no bone-related tissue was available in the GTEx resource. From human genetic association studies, it is not possible to determine if a factor such as RSPO3 exerts local effects within the bone tissue. However, it is possible to determine if the genetic signals that associate with bone health parameters also associate with circulating levels of RSPO3. It should be emphasized that such association does not show that the effect on bone is mediated via circulating levels of RSPO3 as the circulating levels may only reflect the local levels, without being of biological importance as a systemic regulator of local bone metabolism.

We observed that SNP rs3734626, which is highly linked with SNP rs7741021 (*r*^2^ = 0.84; coefficient of linkage disequilibrium measure, *D*′ = 0.99) at the RSPO3 locus, was strongly associated with circulating protein levels of RSPO3 (*P* = 1.8 × 10^−45^; Table 1b)^33^. The association for SNP rs3734626 with the circulating protein levels of RSPO3 was derived from a previous report, demonstrating that this SNP was the most significant cis-protein quantitative trait loci (cis-pQTLs) for RSPO3^33^. The T-allele of rs3734626, associated with reduced fracture risk at any bone site and increased BMD, was associated with increased circulating levels of RSPO3 (Table 1b). Collectively, these human genetic studies indicate that the main fracture signal at the *RSPO3* locus regulates RSPO3 expression and that the fracture-reducing allele associates with both increased RSPO3 expression and BMD.

We next evaluated the impact of rs7741021 on trabecular and cortical bone parameters analyzed separately by pQCT in the tibia in humans^9,34^. The fracture reducing C-allele of rs7741021 was significantly associated with increased trabecular volumetric BMD, while no significant association was observed with cortical volumetric BMD or cortical bone thickness (Table 1a).

As the distal radius is a bone compartment with relatively high proportion of trabecular bone, we next extended the fracture association studies by evaluating the associations for rs7741021 specifically with distal forearm fractures, defined by ICD codes, in the large UK Biobank cohort. The C-allele of rs7741021 was robustly associated with reduced risk of forearm fractures (OR 0.88, 95% confidence intervals (CI) 0.86–0.91 per C-allele; *P* = 9.6 × 10^−14^; Table 1a) in age, age^2^, sex, weight and height adjusted analyses. We also evaluated the association between rs7741021 and hip fractures, defined by ICD codes. The C-allele of rs7741021 was associated with reduced risk of hip fractures (OR 0.94, 95% CI 0.90–0.99 per C-allele; *P* = 4.4 × 10^−3^; Table 1a). The effect size for the association of rs7741021 with distal forearm fractures was significantly larger than the effect sizes for the association of rs7741021 with fractures at any bone site (Table 1a, OR 0.95, 95% CI 0.94–0.96 per C-allele; *P* = 1.6 × 10^−5^; *Z*-test) and hip fractures (*P* = 3.5 × 10^−2^; *Z*-test).

To estimate the impact of RSPO3 on bone parameters and fracture risk, we performed two-sample inverse variance-weighted (IVW) Mendelian randomization (MR) studies. We used either the above described strong cis-pQTL rs3734626 for circulating RSPO3 identified by Emilsson et al. ^33^ (Table 1b) or the linked SNP rs2489623 (*r*^2^ = 0.74, *D*′ = 0.88; Table 1c), a cis-pQTL for circulating RSPO3 (*P* = 3.6 × 10^−28^) identified by Sun et al. in an independent dataset^35^, as genetic instruments for circulating protein levels of RSPO3. These MR analyses indicated that increased circulating RSPO3 was strongly associated with increased trabecular vBMD, and reduced risk of distal forearm fractures, and as expected, with very similar effect sizes for the two genetic instruments (Table 2). However, it should be emphasized that this association might be completely driven by circulating RSPO3 being associated with local RSPO3 levels, and from these human associations studies it is, therefore, not possible to determine possible local effects of factors acting on bone remodeling. Thus, to determine the relative role of circulating RSPO3 and local RSPO3, functional studies in mice with cell-specific inactivation of RSPO3 is required.

**Table 2:** Mendelian randomization studies of the estimated causal associations of circulating RSPO3 level on fractures and bone parameters.

	rs3734626			rs2489623		
**Fractures**	**OR**	**95% CI**	***P*value**	**OR**	**95% CI**	***P*value**
Fracture at any bone site	0.83	(0.80–0.86)	1.6E−20	0.83	(0.79–0.87)	9.0E−15
Distal forearm fractures	0.70	(0.70–0.78)	2.4E−12	0.65	(0.57–0.73)	8.5E−13
Hip fractures	0.84	(0.74–0.97)	1.4E−02	0.88	(0.74–1.03)	1.1E−01
**Bone parameters**	**Beta**	**SE**	***P*value**	**Beta**	**SE**	***P*value**
eBMD	0.21	0.01	2.2E−307	0.23	0.01	1.8E−243
Trabecular vBMD	0.28	0.09	9.4E−04	0.31	0.10	2.5E−03
Cortical vBMD	−0.03	0.05	5.0E−01	0.07	0.06	2.1E−01
Cortical thickness	0.05	0.05	3.7E−01	0.06	0.06	3.4E−01

Inverse variance-weighted (IVW) Mendelian randomization of the estimated causal association of circulating RSPO3 on fractures and bone parameters using rs3734626 (left part) or rs2489623 (right part) as a genetic instrument. Betas in SD (or ORs) are given per SD increase in circulating RSPO3. eBMD = estimated bone mineral density measured by ultrasound in the heel. Trabecular vBMD = Trabecular volumetric BMD as measured by pQCT in the distal tibia metaphysis. Cortical vBMD = Cortical volumetric BMD as measured by pQCT in the tibia diaphysis. Cortical thickness = Cortical thickness measured by pQCT in the diaphysis of tibia. Genetic associations with the exposure (RSPO3) and outcomes (fractures and bone parameter) used in the Mendelian randomization are presented in Table 1b (rs3734626) and Table 1c (rs2489623).The statistical tests were two-sided. No adjustments were made for multiple comparisons.

To exclude reverse causality, wherein BMD influences levels of RSPO3, we performed a bidirectional MR using BMD as the exposure and circulating RSPO3 as the outcome. These studies revealed no evidence of reverse causality (*P* = 0.80).

Colocalization analyses may be used to determine if there is a shared genetic signal between two traits at a specific gene locus. Traditional colocalization analyses require that only one independent association signal exists at a specific locus. Because colocalization inference are very sensitive to small linkage disequilibrium (LD) changes in the tested populations, multiple causal signals presented in the same region may introduce substantial bias^36^. As only one independent association signal was observed for trabecular volumetric BMD at the *RSPO3* locus (Supplementary Fig. 1), we used colocalization analyses to determine if there is a shared genetic signal for circulating RSPO3 and trabecular volumetric BMD at this locus. Bayesian colocalization analyses using COLOC^37^ found that the plasma RSPO3 signal identified by Sun et al. was reasonably well colocalized with the single signal observed for trabecular volumetric BMD at the *RSPO3* locus with a posterior probability of a single shared signal of 72% (posterior probability of the hypothesis 4 that the association with trait 1 and trait 2 has one shared SNP^37^, PP.H4 = 0.72), indicating that there is a single shared genetic signal in the 1MB locus of the *RSPO3* cis-pQTL SNP (rs2489623), affecting both circulating RSPO3 and trabecular volumetric BMD (Supplementary Fig. 1). Since there are multiple independent signals for both estimated BMD (eBMD) and forearm fractures at the *RSPO3* locus, and since we do not have access to any individual level data to assume accurate LD, we did not undertake colocalization analyses for these parameters.

These human genetic studies demonstrate that the strong fracture signal at the *RSPO3* locus has the capacity to regulate *RSPO3* expression, and that increased circulating RSPO3 levels associate with increased trabecular BMD, substantially reduced risk of distal forearm fractures and moderately reduced risk of hip fractures. Based on these human genetic data, we hypothesized that RSPO3 increases trabecular bone mass and thereby reduces fracture risk. To test this hypothesis, we performed extensive mechanistic studies using several conditional *Rspo3*-inactivated mouse models and cultured bone-derived cells.

### *RSPO3* is expressed in osteoprogenitor cells and osteoblasts but not in osteocytes or osteoclasts

**Fig. 1:**
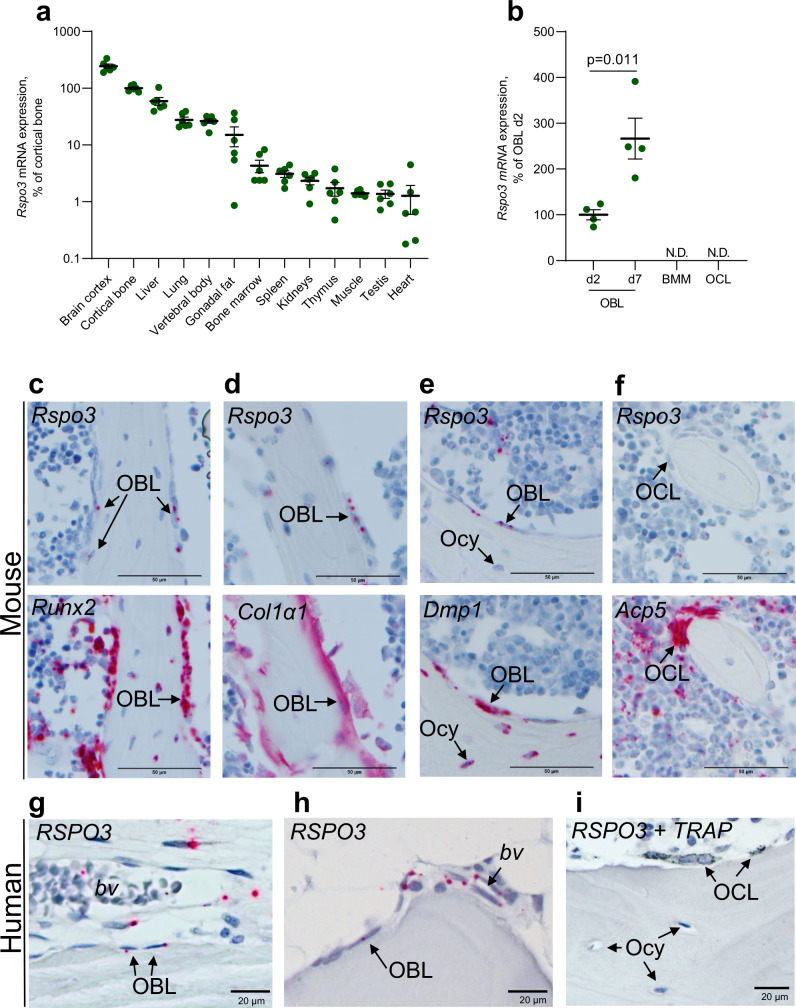
RSPO3 is expressed in osteoblasts but not in osteocytes or osteoclasts. **a***Rspo3* mRNA expression pattern in various tissues of male, wildtype mice (*n* = 6). Mean of individual values are presented as horizontal lines and ±SEM as vertical lines. Data are presented as % of expression in cortical diaphyseal bone. **b** *Rspo3* is expressed in primary calvarial osteoblasts (OBL), but not in bone marrow-derived macrophages (BMM) or RANKL-differentiated osteoclasts (OCL). *Rspo3* gene expression increase from day 2 (d2) to day 7 (d7) in primary calvarial osteoblasts cultured in osteogenic media. Presented as % of expression in osteoblasts day 2. ND=not detectable. Mean of individual values are presented as horizontal lines and ±SEM as vertical lines. Difference in expression between day 2 and 7 was analyzed by two-sided Student’s *t* test, *n* = 4 wells per group. **c–f** Representative in situ hybridization images in mice. Transverse sections across lumbar vertebra 5 in mouse showing the mRNA expression pattern (red) of *Rspo3* (**c**–**f**, upper) and *Runx2* (**c**, lower), *Col1a1* (**d**, lower), *Dmp1* (**e**, lower), and *Acp5/Trap* (**f**, lower). The lower images are consecutive to the upper images. *Rspo3* mRNA could be observed in *Runx2-*expressing (**c**)*, Col1a1-*expressing (**d**), and *Dmp1-*expressing (**e**) osteoblast lineage cells (OBL) on the bone surface. In contrast, *Rspo3* was not detectable in osteocytes (Ocy; **e**), or osteoclasts (OCL; **f**). Scale bar 50 µm. **g–i** Representative in situ hybridization images in humans. Sections from human proximal femur (**g**) and iliac crest (**h**, **i**) confirmed moderate *RSPO3* mRNA expression. Staining for *RSPO3* mRNA (red) were observed in immature osteoblast lineage cells (OBL) on eroded lining surfaces (reversal cells), and on quiescent bone surfaces (bone lining cells), as well as in a subset of cells close to blood vessels (bv; **g**, **h**). No *RSPO3* mRNA expression was observed in osteocytes (Ocy) or TRAP-immunostained osteoclasts (OCL, black; **i**). Scale bar 20 µm. Experiments were repeated one (**a**), two (**g**–**i**) or at least three (**b**–**f**) times. Source data are provided as a Source Data file.

We observed *Rspo3* mRNA expression in several mouse tissues, revealing the highest *Rspo3* expression in brain cortex and diaphyseal cortical bone with intermediate expression in the vertebral body and modest expression in flushed bone marrow (Fig. 1a). *Rspo3* mRNA levels were high in primary calvarial osteoblasts after prolonged culture in osteogenic media (Fig. 1b; Supplementary Fig. 2a). In contrast, no *Rspo3* expression was observed in bone marrow-derived macrophages cultured with M-CSF or osteoclasts differentiated from bone marrow-derived macrophages stimulated with RANKL (Fig. 1b; Supplementary Fig. 2b).

Next, the *RSPO3* expression in mouse and human bone sections was evaluated using chromogenic in situ hybridization (Fig. 1c-i). Assessment of *Rspo3* expression in mouse vertebral bone revealed that *Rspo3* mRNA is expressed in *Runx2*-expressing (Fig. 1c), *Col1a1*-expressing (Fig. 1d) and *Dmp1*-expressing (Fig. 1e) osteoblast-lineage cells on bone surface. In contrast, *Rspo3* expression was not detectable in osteocytes (Fig. 1e) or osteoclasts (Fig. 1f). *Rspo3* expression was also observed in a subset of cells close to blood vessels and adipocytes in the bone marrow (Supplementary Fig. 2c,d). Analyses of human bone sections (Fig. 1g–i) confirmed moderate *RSPO3* mRNA expression in immature osteoblast lineage cells, on eroded lining surfaces (reversal cells) and quiescent bone surfaces (bone lining cells) (Fig. 1g), and in a subset of cells close to blood vessels and adipocytes (Fig. 1h). No staining for *RSPO3* mRNA expression was observed in osteocytes or osteoclasts in human sections (Fig. 1i).

**Fig. 2:**
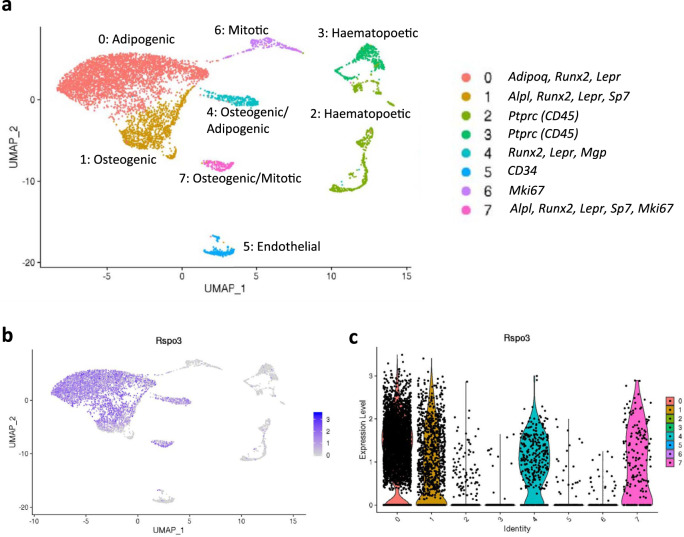
*Rspo3* expression in *Cxcl12-*expressing bone marrow stromal cells. Single cell RNA sequencing analysis of *Cxcl12* expressing bone marrow cells. **a** Uniform Manifold Approximation and Projection (UMAP)-based visualization of major cell clusters (Cluster 0–7) and cluster expressed genes (right). Feature plot (**b**) and violin plot (**c**) of *Rspo3* expression.

Thus, in addition to expression in osteoblast-lineage cells on bone surfaces, *RSPO3* expression was found in a subset of cells close to blood vessels, where mesenchymal skeletal stem cells are located within the bone marrow^38^. To determine *Rspo3* expression specifically in mesenchymal stem cells in the bone marrow, we analyzed two independent single cell RNA-sequencing (scRNA seq) data sets of bone marrow cells. First, scRNA seq analysis of *Cxcl12* expressing bone marrow cells showed that *Rspo3* was expressed in stromal cells expressing both adipogenic and osteoblastic markers (Fig. 2a–c)^39^. These cell clusters also expressed *Lepr* (Supplementary Fig. 3), often used in combination with *Cxcl12* as a marker for mesenchymal stem cells, supporting the notion that *Rspo3* is expressed also in mesenchymal stem cells. No *Rspo3* expression was found in *Cd45*-expressing hematopoietic cell clusters (Fig. 2a–c).

The expression of *Rspo3* in *Cxcl12*^*+*^*Lepr*^*+*^ mesenchymal stem cells was further confirmed in a second recently published scRNA seq dataset (Supplementary Fig. 4)^40^. This dataset includes scRNA seq analysis of cells expressing *Cdh5* (found in vasculature), *Lepr* (perivascular stromal stem cells) and *Col1a1* (osteoblastic cells). Using this dataset, we identified *Rspo3* expression in stromal cells expressing both adipogenic and osteogenic markers (Supplementary Fig. 4). *Rspo3* mRNA was expressed also in pro-osteogenic *Lepr*^+^ cells expressing *Alpl* and in the more mature osteoblast lineage *Col1a1*-expressing cells. No *Rspo3* expression was found in the vascular cell clusters. Collectively, these two scRNA seq data sets of bone marrow cells reveal robust *Rspo3* expression in mesenchymal stem cells expressing both osteogenic and adipogenic markers.

### Osteoblast-derived RSPO3 is the principal source of RSPO3 in bone and is a major regulator of trabecular bone mass

**Fig. 3:**
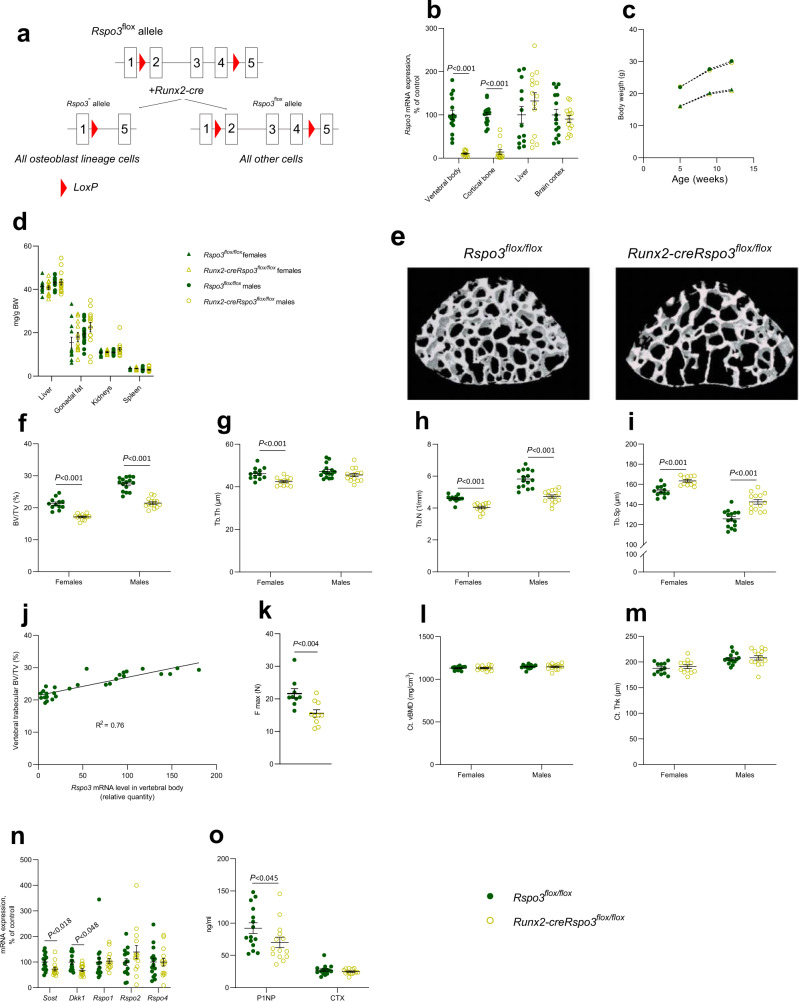
Osteoblast-derived RSPO3 is the principal source of RSPO3 in bone and is a major regulator of trabecular bone mass. **a** Schematic figure of the conditional osteoblast-lineage specific *Rspo3*-inactivated mouse model. **b** mRNA expression analyses of *Rspo3* in vertebral trabecular bone, cortical diaphyseal bone, liver, and brain cortex in male *Runx2-creRspo3*^*flox/flox*^ mice, compared to *Rspo3*^*flox/flox*^ mice. **c** Body weight in *Runx2-creRspo3*^*flox/flox*^ mice compared to *Rspo3*^*flox/flox*^ mice at 5, 9, and 13 weeks-of-age. **d** Weight of liver, gonadal fat, kidneys, and spleen, per body weight (BW) in *Runx2-creRspo3*^*flox/flox*^ mice, and *Rspo3*^*flox/flox*^ mice. **e** Representative 3D µCT images of vertebra L5 in *Runx2-creRspo3*^*flox/flox*^ mouse (left) and *Rspo3*^*flox/flox*^ mouse (right). **f–i** Trabecular bone volume over total volume (BV/TV; **f**), trabecular thickness (Tb.Th; **g**), trabecular number (Tb.N; **h**), and trabecular separation (Tb.Sp; **i**) in vertebra L5 from *Runx2-creRspo3*^*flox/flox*^ mice, compared to *Rspo3*^*flox/flox*^ mice, as measured by µCT. **j** Correlation between *Rspo3* mRNA levels in vertebral body (relative quantity, *x*-axis) and trabecular BV/TV (%, *y*-axis) in male *Runx2-creRspo3*^*flox/flox*^ and *Rspo3*^*flox/flox*^ mice. Variance explained (*r*^2^) is given in the figure. **k** Maximal load at failure (*N*) of vertebra L5 as measured by compression test in 14-week-old male *Runx2-creRspo3*^*flox/flox*^ (*n* = 10) mice and *Rspo3*^*flox/flox*^ (*n* = 9) mice. **l**, **m** Cortical volumetric bone mineral density (Ct.vBMD; **l**) and cortical thickness (Ct.Th; **m**) in femur from *Runx2-creRspo3*^*flox/flox*^ mice, compared to *Rspo3*^*flox/flox*^ mice. **n** mRNA expression of sclerostin (*Sost)*, Dickkopf-1 (*Dkk1)*, and R-spondins 1, -2, and -4 (*Rspo1, Rspo2*, and *Rspo4*) in vertebral body in male *Runx2-creRspo3*^*flox/flox*^ mice, compared to *Rspo3*^*flox/flox*^ mice. **o** Levels of procollagen type I N-terminal propeptide (P1NP, left) and C-terminal type I collagen (CTX, right) in male *Runx2-creRspo3*^*flox/flox*^ mice, compared to *Rspo3*^*flox/flox*^ mice. Unless otherwise stated, the results refer to 13-week-old *Runx2-creRspo3*^*flox/flox*^ mice, males *n* = 14; females *n* = 12, and *Rspo3*^*flox/flox*^ mice, males *n* = 15; females *n* = 12. Individual values are presented in all graphs with the mean presented as horizontal lines and ±SEM as vertical lines. Statistical analyses were performed using two-sided Student’s *t* test. Source data are provided as a Source Data file.

As global *Rspo3*^−*/*−^ mice display early embryonic lethality^29^, and since we observed robust *Rspo3* expression in osteoblast-lineage cells, we first evaluated the impact of osteoblast-derived RSPO3 for trabecular and cortical bone parameters. To this end, we generated a conditional *Rspo3*-inactivated mouse model targeting osteoblasts and osteocytes. A mouse model with exons 2–4 of *Rspo3* flanked by LoxP sites (*Rspo3*^*flox/flox*^, Fig. 3a) was used^41^. To achieve inactivation of *Rspo3* early in the osteoblast lineage, we generated *Runx2-creRspo3*^*flox/flox*^ mice, which express Cre recombinase driven by the *Runx2* promoter, a promoter expressed in early osteoblast lineage cells but not in osteoclasts (Fig. 3a)^42^. *Runx2-creRspo3*^*flox/flox*^ mice had more than 90% reduced *Rspo3* mRNA levels in both vertebral trabecular bone and tibial cortical diaphyseal bone with no reduction in brain cortex or liver compared to *Rspo3*^*flox/flox*^ mice (Fig. 3b). Thus, osteoblast-derived RSPO3 is the principal source of RSPO3 in both trabecular and cortical bones.

*Runx2-creRspo3*^*flox/flox*^ mice were born apparently healthy and had a normal body weight growth at termination at 12 weeks of age and displayed normal weights of several visceral organs (Fig. 3c, d). Consistent with the human genetic findings, CT analyses revealed reduced trabecular bone mass reflected by reduced trabecular bone volume fraction (BV/TV; Fig. 3e, f), trabecular thickness (Fig. 3g), trabecular number (Fig. 3h) and increased trabecular separation (Fig. 3i) in the lumbar vertebra L5 of *Runx2-creRspo3*^*flox/flox*^ in both female and male mice compared to *Rspo3*^*flox/flox*^ mice. Reduced trabecular bone mass in the vertebrae was confirmed by histomorphometric analyses, revealing a 27% (*P* < 0.001) reduction in trabecular BV/TV in *Runx2-creRspo3*^*flox/flox*^ mice (Supplementary Fig. 5a–d). Furthermore, the trabecular BV/TV was positively correlated with *Rspo3* mRNA levels in the vertebral body (*r*^2^ = 0.76, *P* < 0.001; Fig. 3j), supporting the notion that local RSPO3 expression regulates trabecular bone mass. To evaluate whether the decreased trabecular bone mass in mice with *Rspo3* inactivation in osteoblast-lineage cells resulted in decreased bone strength, lumbar vertebra L5 was evaluated using a compression test, demonstrating that the maximal load at failure was significantly decreased in *Runx2-creRspo3*^*flox/flox*^ mice compared with *Rspo3*^*flox/flox*^ mice (Fig. 3k). No significant effect of osteoblast-lineage specific *Rspo3* inactivation was observed on cortical volumetric BMD or cortical thickness in the femur diaphysis (Fig. 3l, m).

Expression analyses in the vertebral body revealed reduced mRNA levels of the two WNT signaling inhibitors *Sost* and *Dkk1* (Fig. 3n), suggesting a compensatory mechanism to counteract additional trabecular bone loss in the adult *Runx2-creRspo3*^*flox/flox*^ mice with life-long inactivation of *Rspo3* in osteoblast-lineage cells. No compensatory regulation of *Rspo1, -2* or -*4* was observed (Fig. 3n).

Circulating levels of the bone formation marker procollagen type I N-terminal propeptide (P1NP; Fig. 3o, left) were reduced while the levels of the bone resorption marker C-terminal type I collagen fragments (CTX; Fig. 3n, right) were unchanged in *Runx2-creRspo3*^*flox/flox*^ mice compared to *Rspo3*^*flox/flox*^ mice. However, neither static nor dynamic histomorphometry of trabecular bone in vertebra L5 or gene expression analyses of vertebral trabecular bone revealed any significant effects on other parameters reflecting bone remodeling in the adult *Runx2-creRspo3*^*flox/flox*^ mice with life-long inactivation of RSPO3, suggesting that a new steady state of bone remodeling had been reached (Supplementary Fig. 5e,f).

**Fig. 4:**
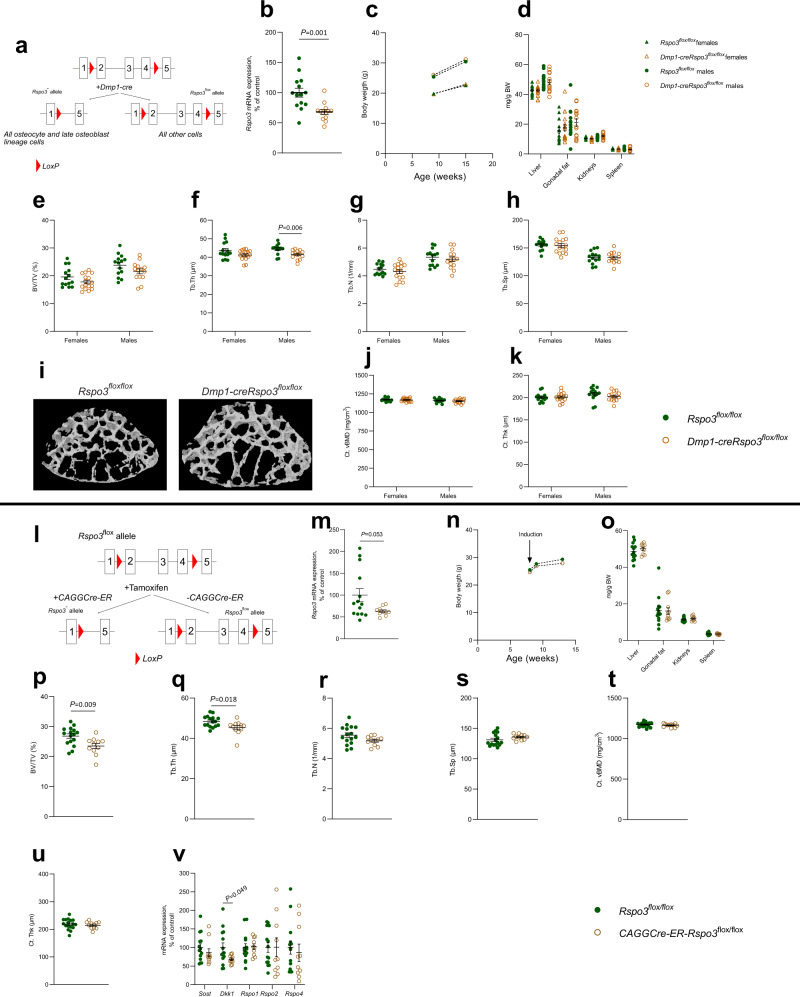
Effects of inactivation of RSPO3 in *Dmp1*-expressing bone cells (a–k) and inducible inactivation of RSPO3 (l–v). **a** Schematic image of the conditional osteocyte/late osteoblast-specific *Rspo3-*inactivated mouse model. **b** mRNA expression analysis of *Rspo3* in vertebral trabecular bone in male *Dmp1cre-Rspo3*^*flox/flox*^ mice, compared to *Rspo3*^*flox/flox*^ mice. **c** Body weight in *Dmp1cre-Rspo3*^*flox/flox*^ mice, and *Rspo3*^*flox/flox*^ mice, at 9 and 15 weeks-of-age. **d** Weight of liver, gonadal fat, kidneys, and spleen per body weight (BW) in *Dmp1-creRspo3*^*flox/flox*^ mice, and *Rspo3*^*flox/flox *^mice. **e–h** Trabecular bone volume over total volume (BV/TV; **e**), trabecular thickness (Tb.Th; **f**), trabecular number (Tb.N; **g**), and trabecular separation (Tb.Sp; **h**) in vertebra L5 from *Dmp1-creRspo3*^*flox/flox*^ mice, compared to *Rspo3*^*flox/flox*^ mice. **i** Representative 3D µCT images of vertebra L5 in *Rspo3*^*flox/flox*^ mouse (left) and *Dmp1creRspo3*^*flox/flox*^ mouse (right). **j** and **k** Cortical volumetric bone mineral density (Ct.vBMD, **j**) and cortical thickness (Ct.Th, **k**) in femur from *Dmp1-creRspo3*^*flox/flox*^ mice, compared to *Rspo3*^*flox/flox*^ mice. **l** Schematic image of the global, tamoxifen-inducible *Rspo3*-inactivated mouse model. **m** mRNA expression analysis of *Rspo3* in vertebral trabecular bone in *CAGGCre-ER-Rspo3*^*flox/flox*^ (*n* = 10) mice, compared to *Rspo3*^*flox/flox*^ (*n* = 14) mice. (**n**) Body weight in male *CAGGCre-ER-Rspo3*^*flox/flox*^ (*n* = 11) mice, and *Rspo3*^*flox/flox*^ (*n* = 17) mice, at 8, 9, and 13 weeks-of-age. **o** Weight of liver, gonadal fat, kidneys, and spleen per body weight in *CAGGCre-ER-Rspo3*^*flox/flox*^ (*n* = 10) mice, and *Rspo3*^*flox/flox*^ mice (*n* = 14). **p–s** Trabecular BV/TV (**p**), trabecular thickness (Tb.Th; **q**), trabecular number (Tb.N; **r**), and trabecular separation (Tb.Sp; **s**) in vertebra L5 from *CAGGCre-ER-Rspo3*^*flox/flox*^ (*n* = 11) mice, compared to *Rspo3*^*flox/flox*^ (*n* = 16) mice. **t**, **u** Cortical volumetric BMD (Ct.vBMD; **t**) and cortical thickness (Ct.Th; **u**) in femur from *CAGGCre-ER-Rspo3*^*flox/flox*^ (*n* = 11) mice, compared to *Rspo3*^*flox/flox*^ (*n* = 17) mice. **v** mRNA expression of sclerostin (*Sost*), Dickkopf-1 (*Dkk1*), and R-spondins 1, -2, and -4 (*Rspo1*, *Rspo2*, and *Rspo4*) in vertebral body in *CAGGCre-ER-Rspo3*^*flox/flox*^ (*n* = 10) mice, compared to *Rspo3*^*flox/flox*^ (*n* = 14) mice. For *Dmp1-creRspo3*^*flox/flox*^ mice, males *n*=14; females *n* = 16, and littermate control *Rspo3*^*flox/flox*^ mice, males *n* = 14; females *n* = 14. Individual values are presented in all graphs with the mean presented as horizontal lines and ±SEM as vertical lines. Statistical analyses were performed using two-sided Student’s *t* test. Source data are provided as a Source Data file.

We next determined if RSPO3 with an impact on trabecular bone is derived from osteoblast precursors/early osteoblasts or from osteocytes/late osteoblasts. To this end, we compared the phenotype of *Runx2-creRspo3*^*flox/flox*^ mice, having inactivation of *Rspo3* in all osteoblast-lineage cells, with the phenotype of a mouse model with *Rspo3* inactivated specifically in *Dmp1*-expressing osteocytes and late osteoblasts. *Rspo3*^*flox/flox*^ mice were mated with *Dmp1*-cre mice expressing Cre recombinase specifically in late osteoblasts and osteocytes^43^, hereafter called *Dmp1cre-Rspo3*^*flox/flox*^ mice (Fig. 4a). *Dmp1cre-Rspo3*^*flox/flox*^ mice displayed a moderate reduction of *Rspo3* mRNA levels in the vertebral body compared with *Rspo3*^*flox/flox*^ mice (−32%; *P* < 0.001; Fig. 4b). The substantially less pronounced reduction of *Rspo3* mRNA levels in the vertebral body of *Dmp1cre-Rspo3*^*flox/flox*^ mice compared with the substantial reduction (>90%, *P* < 0.001) in *Runx2-creRspo3*^*flox/flox*^ mice demonstrates that osteoblasts, and not osteocytes, are the major source of RSPO3 in vertebral trabecular bone (Figs. 3b and 4b).

*Dmp1cre-Rspo3*^*flox/flox*^ mice were born apparently healthy and had a normal body weight growth at termination at 15 weeks of age and displayed normal weights of several visceral organs (Fig. 4c, d). CT analyses revealed modestly reduced trabecular bone volume fraction (−9.3%; BV/TV, Fig. 4e–i) in *Dmp1cre-Rspo3*^*flox/flox*^ mice compared with *Rspo3*^*flox/flox*^ mice, reaching statistical significance in a two-way ANOVA considering genotype and sex (*P* = 0.027 for genotype). The reduction of trabecular bone volume fraction, however, was substantially more pronounced in the *Runx2-creRspo3*^*flox/flox*^ mice compared with the reduction observed in *Dmp1cre-Rspo3*^*flox/flox*^ mice (Figs. 3f, 4e), demonstrating that RSPO3 with an impact on trabecular bone is mainly osteoblast- and not osteocyte-derived. As expected, no effect on cortical bone density or cortical bone thickness was observed in the *Dmp1cre-Rspo3*^*flox/flox*^ mice (Fig. 4j, k).

### Inducible inactivation of *Rspo3* reduces trabecular bone volume fraction

Although very informative, the studies using life-long osteoblast-lineage specific *Rspo3* inactivation cannot separate between early developmental effects of RSPO3 and its effects on adult bone metabolism. To evaluate the effect of RSPO3 specifically on adult bone homeostasis and the underlying mechanism, before a compensatory new steady state of bone remodeling has been reached, we developed a mouse model with tamoxifen-inducible *Rspo3* inactivation in adult mice. To this end, we bred *Rspo3*^*flox/flox*^ mice with *CAGGCre-ER* transgenic mice expressing a tamoxifen-inducible Cre-mediated recombination system (Fig. 4l)^44^. We have previously shown that *CAGGCre-ER* mice do not have a skeletal phenotype^45^. All mice evaluated were treated with the same tamoxifen regime and the phenotype of *CAGGCre-ER-Rspo3*^*flox/flox*^ and *Rspo3*^*flox/flox*^ mice were compared (Fig. 4l). The *Rspo3* mRNA levels were lower (−38%, *P* = 0.05) in the vertebral body of tamoxifen-treated *CAGGCre-ER-Rspo3*^*flox/flox*^ mice compared with tamoxifen-treated *Rspo3*^*flox/flox*^ mice (Fig. 4m). To determine the skeletal phenotype in the inducible *Rspo3* knockout mice, 8-week-old *Rspo3*^*flox/flox*^ and *CAGGCre-ER-Rspo3*^*flox/flox*^ mice were treated with tamoxifen and the phenotype was evaluated 38 days after the first tamoxifen dose. The inducible inactivation of *Rspo3* did not affect body weight or weights of several visceral organs (Fig. 4n,o). Analyses of the vertebral trabecular bone volume fraction revealed a significant reduction both when evaluated using CT (BV/TV; −12.3%, *P* = 0.009, Fig. 4p–s) and histomorphometry (−18.0%, *P* = 0.038; Supplementary Fig. 6a–d) in tamoxifen-treated *CAGGCre-ER-Rspo3*^*flox/flox*^ mice compared with tamoxifen-treated *Rspo3*^*flox/flox*^ mice. Inducible *Rspo3* inactivation did not affect cortical bone density or cortical bone thickness (Fig. 4t, u). Expression analyses in the vertebral body revealed that inducible *Rspo3* inactivation reduced *Dkk1* mRNA levels (−32%, *P* = 0.049; Fig. 4v), suggesting a compensatory mechanism to counteract additional trabecular bone loss. Dynamic histomorphometry of vertebral trabecular bone demonstrated a 31% reduction of trabecular bone formation rate per tissue volume (*P* = 0.047, Supplementary Fig. 6e) and similar effect sizes were observed for number of osteoblasts per trabecular bone perimeter (−32%, *P* = 0.088; Supplementary Fig. 6f) and for levels of the circulating bone formation marker P1NP (−20%, *P* = 0.067; Supplementary Fig. 6g) in tamoxifen-treated *CAGGCre-ER-Rspo3*^*flox/flox*^ mice compared with tamoxifen-treated *Rspo3*^*flox/flox*^ mice. In contrast, there was no indication of bone resorption being affected in mice with inducible *Rspo3* inactivation (Supplementary Fig. 6f, g). To further explore the role of osteoblast-derived RSPO3 on bone formation, we performed extensive studies using different osteoblast cell cultures.

### Osteoblast-derived RSPO3 increases osteoblast proliferation and differentiation

In our first attempts to assess the role of cell-autonomous RSPO3 for osteoblast differentiation we used calvarial osteoblasts isolated from newborn *Rspo3*^*flox/flox*^ and *Runx2-creRspo3*^*flox/flox*^ mice. The cells were incubated for 4 days in culture flasks to expand cell numbers and subsequently seeded in multiwells for 3 days. *Rspo3* mRNA was moderately lower (−59%; *P* < 0.01) in cells from *Runx2-creRspo3*^*flox/flox*^ mice compared with cells from *Rspo3*^*flox/flox*^ mice and this was associated with a significant decrease of *Alpl* mRNA (−61%, *P* = 0.011; Supplementary Fig. 7a).

**Fig. 5:**
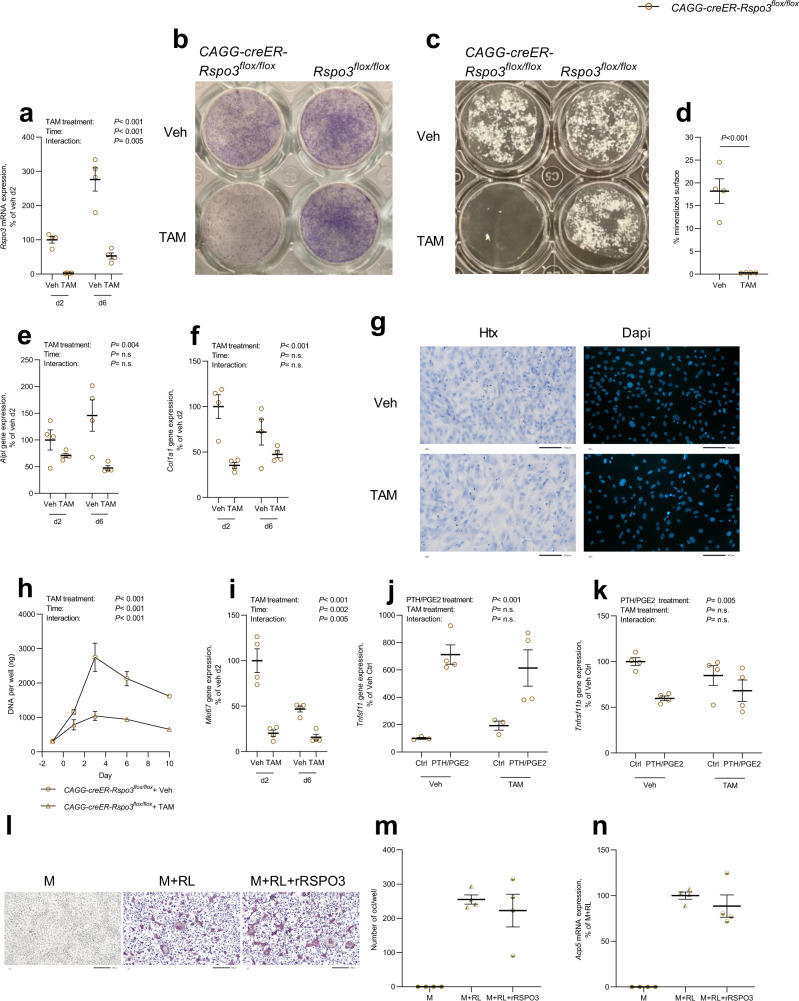
Osteoblast-derived RSPO3 increases osteoblast proliferation and differentiation. **a***Rspo3* mRNA expression in primary calvarial osteoblasts (cOBL) isolated from *CAGGCre-ER-Rspo3*^*flox/flox*^ mice cultured for 2 and 6 days (d) with or without prior *Rspo3* inactivation using tamoxifen (TAM). Veh=vehicle. **b** Representative photos of alkaline phosphatase staining of *CAGGCre-ER-Rspo3*^*flox/flox*^ and *Rspo3*^*flox/flox*^ cOBL cultured for 6 days after veh treatment or *Rspo3* inactivation. **c**, **d** Representative photos (**c**) and quantification (**d**) of mineralized noduli in cultures of *CAGGCre-ER-Rspo3*^*flox/flox*^ and *Rspo3*^*flox/flox*^ cOBL cultured for 14 days after veh treatment or *Rspo3* inactivation. **e**, **f** mRNA expression analysis of *Alpl* (**e**) and *Col1a1* (**f**) in *CAGGCre-ER-Rspo3*^*flox/flox*^ cOBL cultured for 2 and 6 days with or without prior *Rspo3* inactivation. **g** Haematoxylin (Htx) and Dapi staining of *CAGGCre-ER-Rspo3*^*flox/flox*^ cOBL cultured for 3 days with or without prior *Rspo3* inactivation. Scale bar 100 μm. **h** Amount of DNA per well in *CAGGCre-ER-Rspo3*^*flox/flox*^ cOBL at the time of TAM addition (day −1), directly after removal of TAM (day 0) and after culture for 1, 3, 6, and 10 days. **i** *Mki67* mRNA expression in *CAGGCre-ER-Rspo3*^*flox/flox*^ cOBL cultured for 2 days after *Rspo3* inactivation. **j**, **k** mRNA expression of *Tnfsf11* (**j**) and *Tnfrsf11b* (**k**) in *CAGGCre-ER-Rspo3*^*flox/flox*^ cOBL cultured with or without PTH and PGE2 for 5 days with or without prior *Rspo3* inactivation. *n* = 3–4 wells. **l–n** TRAP staining (**l**), counting (**m**), and *Acp5* mRNA expression (**n**) of bone marrow macrophages cultured in M-CSF (M) or in M-CSF and RANKL (RL) to induce osteoclastogenesis in the absence or presence of recombinant RSPO3 (rRSPO3). Scale bar 200 μm. Individual values are presented in all graphs with the mean presented as horizontal lines and ±SEM as vertical lines. If not otherwise stated, *n* = 4 wells per group. Experiments were repeated two (**g**, **j**–**n**) or at least three (**a**–**f**, **h**, **i**) times. For **a**, **e**, **f**, **h**, two-way ANOVA was used to determine the overall effect of *Rspo3* inactivation by treatment of TAM or veh, time, as well as their interaction. For **j**, **k**, two-way ANOVA was used to determine the overall effect of PTH/PGE2 treatment, *Rspo3* inactivation by treatment of TAM or veh, as well as their interaction. When only the effect of *Rspo3* inactivation was evaluated, two-sided Student’s *t* was used. Source data are provided as a Source Data file.

To achieve a more efficient *Rspo3* inactivation in calvarial osteoblasts, we used cells isolated from *CAGGCre-ER-Rspo3*^*flox/flox*^ mice treated with tamoxifen in vitro, resulting in an almost complete inactivation of *Rspo3* mRNA expression (Fig. 5a), whereas tamoxifen-treated cells from *Rspo3*^*flox/flox*^ mice had unaltered *Rspo3* mRNA levels (Supplementary Fig. 7b). When these calvarial osteoblasts were cultured in osteogenic medium it was observed that inactivation of *Rspo3* resulted in decreased osteoblast differentiation as assessed by substantially decreased ALP staining in culture using cells from tamoxifen-treated *CAGGCre-ER-Rspo3*^*flox/flox*^ mice as compared to genotypically identical cells not treated with tamoxifen, or with cells from *Rspo3*^*flox/flox*^ mice incubated in the absence or presence of tamoxifen (Fig. 5b). Importantly, osteoblasts from tamoxifen-treated *CAGGCre-ER-Rspo3*^*flox/flox*^ mice had almost completely lost the capability to form mineralized noduli, in contrast to cells from the same genotype not treated with tamoxifen (Fig. 5c, d), or cells from *Rspo3*^*flox/flox*^ mice with or without tamoxifen (Fig. 5c, Supplementary Fig. 7c). The effects on ALP activity and bone noduli formation were associated with lower expression of *Alpl* and *Col1a1* mRNA in tamoxifen-treated *CAGGCre-ER-Rspo3*^*flox/flox*^ cells (Fig. 5e,f) as compared to the absence of effect by tamoxifen in cells from *Rspo3*^*flox/flox*^ mice (Supplementary Fig. 7d, e).

Deletion of *Rspo3* in tamoxifen-treated cells from *CAGGCre-ER-Rspo3*^*flox/flox*^ mice resulted in less dense cell cultures due to decreased cell numbers as demonstrated by decreased amounts of DNA, an effect absent in tamoxifen-treated cells from *Rspo3*^*flox/flox*^ mice or cells from *Rspo3*^*flox/flox*^ mice with or without tamoxifen (Fig. 5g, h, Supplementary Fig. 7f). Reduced cell numbers were due to an effect on cell proliferation as demonstrated by lower mRNA expression of *Mki67*, a well-established marker for cell proliferation (Fig. 5i, Supplementary Fig. 7g). Collectively, these studies demonstrate that RSPO3 enhances osteoblast proliferation and differentiation in a cell-autonomous manner.

In agreement with the in vivo observations, RSPO3 did not affect osteoclast formation as demonstrated by the observations that the osteoclastogenic response to PTH in the calvarial osteoblasts (increased *Tnfsf11* and decreased *Tnfrsf11b* mRNA) was not affected by deletion of *Rspo3* (Fig. 5j, k, Supplementary Fig. 7h, i), nor did addition of recombinant RSPO3 protein affect osteoclast formation induced by RANKL in bone marrow macrophage cultures (Fig. 5l–n).

### RSPO3 increases osteoblast proliferation and differentiation through WNT-β-catenin signaling

**Fig. 6:**
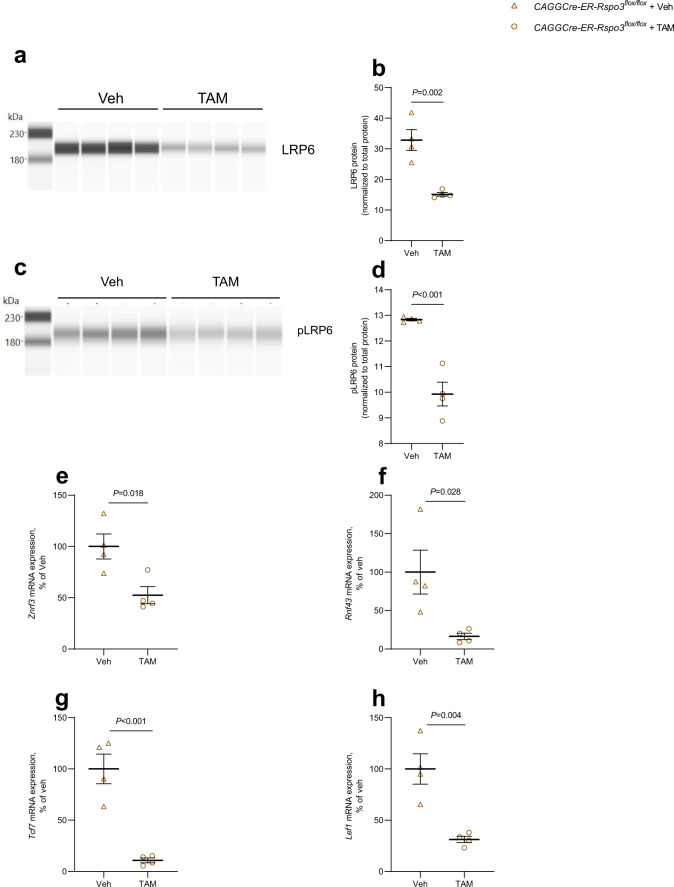
Deletion of RSPO3 in osteoblasts decreased LRP6 and E3 ligases important for WNT canonical signaling. **a**, **b** LRP6 protein analysis in primary calvarial osteoblasts isolated from *CAGGCre-ER-Rspo3*^*flox/flox*^ mice cultured in osteogenic media for 1 day with or without prior *Rspo3* inactivation using tamoxifen (TAM). Lane view from the software in the capillary-based electrophoresis immunodetection system (**a**) and protein levels (**b**) normalized to total protein. **c**, **d** pLRP6 protein analysis in primary calvarial osteoblasts isolated from *CAGGCre-ER-Rspo3*^*flox/flox*^ mice cultured in osteogenic media for 1 day with or without prior *Rspo3* inactivation using tamoxifen (TAM). Lane view from the software in the capillary-based electrophoresis immunodetection system (**c**) and protein levels (**d**) normalized to total protein. **e–h** mRNA expression analyses of *Znrf3* (**e**), *Rnf43* (**f**), *Tcf7* (**g**) and *Lef1* (**h**) in primary calvarial osteoblasts isolated from *CAGGCre-ER-Rspo3*^*flox/flox*^ mice cultured in osteogenic media for 6 days with or without prior *Rspo3* inactivation using TAM. Veh=vehicle. Individual values are presented in all graphs with the mean presented as horizontal lines and ±SEM as vertical lines. *n* = 4 wells per group. Experiments were repeated one (**e**, **f**), two (**a**, **b**, **g**, **h**) or at least three (**c**, **d**) times. Statistical analyses were performed using two-sided Student’s *t* test. Source data are provided as a Source Data file.

Through their Furin-like domain 1, RSPOs bind to extracellular domains of the E3 ligases ZNRF3/RNF43, which are important for ubiquitination and proteasomal degradation of LRP6 and Frizzleds^46^. When the ternary complex of RSPOs, LGRs and ZNRF3/RNF43 is formed, ZNRF3/RNF43 is endocytosed and degraded, resulting in decreased breakdown of Frizzleds and LRP6, and consequently to increased sensitivity to WNT canonical signaling^47,48^. We found that deletion of *Rspo3* in tamoxifen-treated osteoblasts from *CAGGCre-ER-Rspo3*^*flox/flox*^ mice resulted in decreased amounts of total LRP6 as well as pLRP6 (Fig. 6a–d). In contrast, tamoxifen treatment did not affect total amounts of LRP6 in osteoblasts from *Rspo3*^*flox/flox*^ mice (Supplementary Fig. 8a, b). Increased WNT canonical signaling is associated with enhanced mRNA expression of negative regulators such as the ligases *Znrf3* and *Rnf43*^47,48^. We noticed that the mRNA expression of *Znrf3* and *Rnf43* was decreased in tamoxifen-treated osteoblasts from *CAGGCre-ER-Rspo3*^*flox/flox*^ mice (Fig. 6e, f), whereas tamoxifen did not affect their expression in osteoblasts from *Rspo3*^*flox/flox*^ mice (Supplementary Fig. 8c, d). These observations suggest that deletion of *Rspo3* in the osteoblasts results in decreased WNT canonical signaling. To directly assess if downregulation of LRP6 and decreased phosphorylation of LRP6 reduced WNT canonical intracellular signaling, we analyzed the mRNA expression of *Tcf7* and *Lef1*, two well-known WNT canonical target genes^49^. It was found that the mRNA expressions of both *Tcf7* and *Lef1* were significantly decreased in osteoblasts from *CAGGCre-ER-Rspo3*^*flox/flox*^ mice treated with tamoxifen (Fig. 6g, h). In contrast, tamoxifen treatment of osteoblasts from *Rspo3*^*flox/flox*^ mice did not affect *Tcf7* or *Lef1* mRNA expression (Supplementary Fig. 8e, f).

## Discussion

Bone fractures are a major public health concern. Recent large-scale human genetic studies have identified 15 loci associated with fractures at any bone site and the understanding of the causal genes and underlying mechanisms for these fracture signals may result in improved prevention and/or treatment of patients with high fracture risk^3,8^. A major genetic determinant for fractures at any bone site is located at the *RSPO3* locus. We, herein, used a combination of human genetic studies and extensive functional mechanistic studies to identify the causal gene and its mechanism for the fracture signal at the *RSPO3* locus. Human genetic studies revealed that the strong fracture association signal at the *RSPO3* locus has the capacity to regulate *RSPO3* expression, and that increased circulating RSPO3 levels associate with increased trabecular BMD and substantially reduced risk of distal forearm fractures. Studies using several different conditional *Rspo3*-inactivated mouse models revealed that osteoblast-derived RSPO3 is the principal source of RSPO3 in bone and a major regulator of trabecular bone volume fraction in mice. Further functional studies revealed that osteoblast-derived RSPO3 improves bone strength in vertebrae of mice. Mechanistic studies showed that osteoblast-derived RSPO3 increases osteoblast proliferation and differentiation via cell-autonomous effects, involving WNT canonical signaling. Given that the RSPO3 locus confers amongst the largest effects on fracture risk in the human genome, these findings point to a protein, whose effects may be a major contributor to fracture risk in humans.

Several previous studies have reported that genetic determinants at the *RSPO3* locus are robustly associated with fractures at any bone site, BMD as measured by DXA at the hip and/or spine, and estimated BMD as determined by ultrasound in the heel^3,8,50–55^. Peripheral CT, but not DXA or ultrasound, can differentiate between the cortical and trabecular bone compartments. We extended previous human genetic studies by showing that the most prominent fracture signal at the *RSPO3* locus was significantly associated with trabecular volumetric BMD as measured by peripheral CT in the distal metaphyseal region of tibia, while no clear association was observed with cortical bone parameters in the diaphyseal region of tibia. These findings suggest that the main fracture signal at the *RSPO3* locus has an impact on fracture risk mainly via an effect on the trabecular bone mass^9,34^. An effect mediated via trabecular bone mass was supported by our findings from larg -scale bone site-specific fracture association analyses, revealing a pronounced association for the genetic signal at the *RSPO3* locus with fractures at the distal forearm, a bone site dependent on trabecular bone.

These human genetic studies, however, did not determine if RSPO3 is the causal gene. Our first evidence of RSPO3 being the causal gene was the observations that the prominent fracture association signal at the *RSPO3* locus was associated with *RSPO3* mRNA levels in both subcutaneous adipose tissue and cultured fibroblasts and circulating RSPO3 protein levels in two independent cohorts^33,35^. Importantly, the fracture reducing allele at the *RSPO3* locus was associated with increased RSPO3 expression both at the mRNA and protein levels and increased trabecular volumetric BMD in the distal tibia. Based on these human genetic data, we hypothesized that RSPO3 increases trabecular bone mass and thereby reduces fracture risk. To test this hypothesis, we performed extensive mechanistic studies using several conditional *Rspo3*-inactivated mouse models and cultured bone-derived cells.

In descriptive studies, we first demonstrated that substantial RSPO3 expression was observed in both cortical and trabecular bone. Detailed cell-specific expression analyses using chromogenic in situ hybridization, single cell RNA sequencing of bone marrow cells and cultured bone-derived cells, revealed RSPO3 expression in osteoprogenitor cells and osteoblasts but not in osteocytes or osteoclasts in both humans and mice. The single cell RNA sequencing data showed RSPO3 expression in mesenchymal stem cells in the bone marrow, supporting previous studies describing RSPO3 expression in different types of stem cells^56^. These descriptive findings were verified by functional studies using mouse models with inactivation of *Rspo3* in osteoblast-lineage cells. To determine the source of RSPO3 in bone, we developed and compared conditional *Rspo3*-inactivated mouse models with inactivation of *Rspo3* early in both osteoblasts and osteocytes (*Runx2-cre*) or in late osteoblasts and osteocytes (*Dmp1-cre*). *Rspo3* mRNA levels in bone were almost completely abolished in the mice with inactivation of *Rspo3* early in both osteoblasts and osteocytes while only marginal reduction was observed in mice with inactivation only in late osteoblasts and osteocytes. These findings demonstrate that osteoblasts and not osteocytes or osteoclasts are the major source of RSPO3 in both trabecular and cortical bone.

Importantly, inactivation of *Rspo3* in both early osteoblasts and osteocytes resulted in a marked reduction of the trabecular bone volume fraction in the vertebrae, whereas inactivation of *Rspo3* in late osteoblasts and osteocytes only marginally reduced the trabecular bone volume fraction, demonstrating that osteoblast-derived, but not osteocyte-derived RSPO3, is a major regulator of trabecular bone mass. Furthermore, the trabecular bone volume fraction was strongly directly correlated with *Rspo3* mRNA levels in the vertebral body, supporting the notion that local *Rspo3  * expression regulates trabecular bone mass in mice.

Although informative, the studies using life-long osteoblast-specific RSPO3 inactivation could not exclude early developmental effects, confounding the adult phenotype. Therefore, to evaluate the effect of RSPO3 on adult trabecular bone homeostasis, we developed a mouse model with tamoxifen-inducible *Rspo3* inactivation in adult mice. A reduction of trabecular bone volume fraction was observed 5.5 weeks after the inducible *Rspo3* inactivation in adult mice, excluding confounding developmental effects and demonstrating that endogenous RSPO3 exerts crucial effects on trabecular bone homeostasis in adult mice. In contrast, no effect on cortical bone thickness or cortical volumetric BMD was observed in mice with life-long osteoblast-lineage specific *Rspo3* inactivation or global adult inducible inactivation of *Rspo3*. These findings are in accordance with the human genetic finding in the present study, demonstrating that the most prominent fracture signal at the *RSPO3* locus was associated with trabecular volumetric BMD, but not with cortical bone thickness or cortical volumetric BMD. Collectively, these findings indicate that RSPO3 mainly affects trabecular bone mass in both humans and mice.

It is well established that trabecular and cortical bone may respond differently to treatments^1^, and that WNT signaling has the capacity to regulate both the trabecular and the cortical bone compartments^10,17–19,57,58^. Previous studies have demonstrated that osteoblast-derived WNT16 is the major known cortical bone-specific WNT and we recently reported that the secreted WNT lipase NOTUM is a major regulator specifically of cortical bone mass with no effect on trabecular bone^10,20,45,59^. In contrast, we, herein, report that the extracellular WNT-signaling modulator RSPO3 mainly regulates trabecular bone mass. Similarly, WNT10b protects against age-dependent trabecular bone loss^19^. Thus, the data presented in the present study corroborate the clinical findings and suggest that the cortical and trabecular compartments are indeed biologically different, their homeostasis being independently regulated by different signaling molecules, all of which are involved in the regulation of bone mass^10^.

Mechanistic studies using a mouse model with inducible inactivation of *Rspo3* in adult mice, and inducible *Rspo3* inactivation in cultured osteoblasts, collectively revealed that osteoblast-derived RSPO3 increases osteoblast proliferation and differentiation in a cell-autonomous manner. A stimulatory effect in vivo of RSPO3 on bone formation is supported by a recent phase 1 clinical trial, evaluating the safety of different doses of an anti-RSPO3 antibody (OMP-131R10/rosmantuzumab) in patients with advanced solid tumors. High doses of the anti-RSPO3 antibody reduced serum levels of the bone formation marker P1NP in a majority of the evaluated subjects, indicating that RSPO3 enhances bone formation also in humans (https://publications.oncomed.com/Ph-1ab-OMP-131R10-anti-RSPO3-adv-solid-tumor-prev-treat-met-CRC-2016-EORTC-NCI-AACR.pdf*Downloaded 2020.11.24*).

RSPOs are potent enhancers of WNT signaling by making up a ternary complex with LGR receptors 4–6 and the E3 ubiquitin ligases ZNRF3/RNF43, through their furin-like domains 2 and 1, respectively^30,31,46^. A role of LGR4 for bone mass is suggested by the observations that a rare nonsense mutation within the *LGR4* gene was reported to be strongly associated with low BMD and with osteoporotic fractures in Icelandic individuals^60^, and that global deletion of *Lgr4* in mice results in reduced vertebral trabecular bone volume due to reduced bone formation^61^, suggesting that RSPO3 might influence bone formation in trabecular bone via activation of LGR4. Formation of the ternary complex results in membrane clearance of ZNRF3/RNF43 and thereby decreased proteasomal degradation of the WNT receptors Frizzleds and the Frizzled co-receptor LRP6^47,48^, known to be important in WNT canonical signaling^49^. In a feedback loop, the mRNA expressions of *Znrf3* and *Rnf43* are increased^47,48^. We found that LRP6 protein was substantially decreased, and that the mRNA expressions of *Znrf3* and *Rnf43* were downregulated in osteoblasts in which *Rspo3* had been deleted, suggesting that WNT canonical signaling was decreased as a consequence of *Rspo3* deletion. Interestingly, LRP6 has previously been reported to be important for trabecular bone and osteoblast differentiation^62^. The fact that the mRNA expressions of *Tcf7* and *Lef1*, two classical signature genes induced by WNT canonical signaling^49^, were decreased in osteoblasts with deletion of *Rspo3*, provides direct evidence that WNT canonical signaling is enhanced by RSPO3. These findings indicate that RSPO3 stimulates osteoblast proliferation and differentiation through enhanced WNT canonical signaling.

Similar to our present observations for RSPO3, it has been reported that also RSPO1 and RSPO2 can stimulate osteoblast differentiation in vitro and that deletion of *Rspo2* in osteocalcin-expressing osteoblasts reduces trabecular bone mass due to decreased bone formation^22,63^. However, human genetic studies have not demonstrated any strong association between genetic variants at the *RSPO1* or *RSPO2* loci and bone mass or fracture susceptibility, suggesting that RSPO3 is the major RSPO-regulating bone mass and fracture susceptibility in humans. A previous report claimed that RSPO3 regulates the differentiation of human adipose-derived stem cells via an activation of LGR4^64^. However, one effect of RSPO3-LGR4 activation in adipose-derived stem cells in vitro in that study was to inhibit their osteogenic potential while a robust stimulatory effect of RSPO3 on osteoblast differentiation and proliferation of osteoprogenitors/osteoblasts both in in vivo and in vitro was observed in the present study. Differential effects of RSPO3 with capacity to both increase and reduce adipogenesis, depending on the origin of adipose progenitors, have recently been described for abdominal-derived and gluteal-derived adipose progenitors^65^. Thus, RSPO3 may either enhance or reduce proliferation and/or differentiation in a cell-type specific and differentiation stage-dependent manner. In the present study, we observed RSPO3 expression in adipocytes within the bone marrow, in both mouse and human, as well as in scRNA seq of bone marrow adipogenic stromal cells. However, the functional role of RSPO3 in bone marrow adipocytes needs to be evaluated in future studies.

In conclusion, using a combination of human genetic studies and extensive functional mechanistic studies, we identified the causal gene and its mechanism for the prominent fracture association signal at the *RSPO3* locus. We provide human genetic evidence that the fracture reducing allele at that locus increases RSPO3 expression, circulating RSPO3 level and trabecular bone mass and that it mainly reduces the risk of distal forearm fractures. The underlying mechanism includes a cell-autonomous effect of RSPO3 on osteoblast proliferation and differentiation.

## Methods

### Human genetic association studies

#### Look ups of fracture signals at the *RSPO3* locus in different available data sets

*Fractures at any bone site:* Association with fractures at any bone site for the most significant SNP at the *RSPO3* locus (rs7741021) is available in Table S4 from Morris et al.^8^ while the associations for the other evaluated SNPs at this locus (rs3734626 and rs2489623) are taken from the GWAS summary statistics for fractures at any bone site, available at the GEFOS website http://www.gefos.org.

*Estimated BMD (eBMD) at the heel analyzed by ultrasound***:** Associations with eBMD for the evaluated SNPs at the *RSPO3* locus are taken from the GWAS summary statistics from Morris et al.^8^, available at the GEFOS website http://www.gefos.org.

*Trabecular vBMD, cortical vBMD and cortical thickness as analyzed by peripheral quantitative computed tomography (pQCT):* Associations for the evaluated SNPs at the *RSPO3* locus with trabecular vBMD in the distal metaphyseal region of tibia and cortical vBMD in the tibia diaphyseal region are taken from the GWAS meta-analyses summary statistics from Paternoster et al.^34^, while the association with cortical bone thickness in the tibia diaphyseal region are taken from Zheng et al.^9^.

*eQTLs:* Associations with eQTLs for the three SNPs at the *RSPO3* locus were evaluated using the GTEx Portal https://www.gtexportal.org/home/

*cis-pQTL:* The associations for the most significant cis-protein QTLs (cis-pQTLs) for RSPO3 were taken from two independent data sets, including rs3734626 from Emilsson et al. (Supplemental Table 13)^33^ and rs2489623 from Sun et al. (Supplementary Table 4)^35^.

#### New associations analyses of candidate SNPs at the *RSPO3* locus with distal forearm fractures and hip fractures

Our new analyses of associations between candidate SNPs at the *RSPO3* locus with distal forearm fractures (defined by ICD10 codes S52.5 and S52.6; *N* = 7324 distal forearm fracture cases) and hip fractures (defined by ICD10 codes S72.0, S72.1 and S72.2; *N* = 4035 hip fracture cases) were performed using data from the UK Biobank study (http://www.ukbiobank.ac.uk). Briefly, the UK Biobank is a large prospective cohort study of approximately a half-million adult (ages 40–69 years) participants living in the United Kingdom, recruited from 22 centers across the United Kingdom in 2006–2010^66^. We included 438,756 participants of white European descent with valid data on the candidate SNPs at the *RSPO3* locus, the two fracture outcomes (distal forearm fractures and hip fractures) and relevant covariates (age, sex, height, weight). The UK Biobank has ethical approval from the North West Multi-Centre Research Ethics Committee (application no. 16/NW/0274), and informed consent was obtained from all participants. The present research was approved by the UK Biobank Research and Access Committee (application no. 51784).

Associations between the three candidate SNPs at the *RSPO3* locus and the two fracture outcomes (distal forearm fractures and hip fractures) were performed using BOLT-LMM^67^. Briefly, this method uses a linear mixed model to account for relatedness and population structure. Fracture risk was corrected for age, age^2^, height, weight, sex, genotyping array and PC1 + … + PC20 in logistic regression models. Individuals were excluded based on unusually high heterozygosity or >5% missing genotype rate, a mismatch between self-reported and genetically inferred sex.

#### Mendelian randomization (MR)

We performed MR analyses to determine the causal associations of RSPO3 on fracture risk and bone parameters using inverse variance-weighted (IVW) MR. The most significant cis-pQTL, either rs3734626 from Emilsson et al.^33^, or rs2489623 from the independent data set of Sun et al.^35^, was used as genetic instrument for circulating RSPO3. Genetic associations with the exposure (RSPO3) and outcomes (fractures and bone parameter) used in the Mendelian randomization are presented in Table 1b (rs3734626) and Table 1c (rs2489623). As genetic variants are randomly distributed at birth, they are unaffected by confounders. We then regressed the association of the cis-pQTL SNP on the outcome measure, weighing its effect by the magnitude of its effect upon the corresponding exposure.

In order to reduce potential horizontal pleiotropy, we used only cis-SNPs for serum RSPO3 levels. These cis-SNPs for RSPO3 were defined in the published GWAS as the lead SNPs within 300 kB, in the data set of Emilsson et al.^33^, or 1 MB, in the independent data set of Sun et al.^35^, of the *RSPO3* gene. Neither of the cis-SNPs at the *RSPO3* locus from the two different sources were associated with any other circulating protein levels included in the extensive circulating proteomic analyses, reducing the risk of potential horizontal pleiotropy. To assess the possibility of reverse causality that bone mass regulates circulating RSPO3, a bidirectional MR using eBMD as the exposure and circulating RSPO3 from Sun et al*.*^35^ as the outcome was performed, using 1107 LD-independent SNPs from Morris et al.^8^ as instrumental variables for eBMD, followed by IVW to meta-analyze their combined effect on the RSPO3 levels.

#### Colocalization

We tested for colocalization of the genetic signal for circulating RSPO3 and trabecular volumetric eBMD using colocalization analyses, which assesses potential confounding by linkage disequilibrium. A stringent Bayesian analysis implemented in COLOC R package was performed to estimate the posterior probability (PP) that the same causal signal in the 1 MB genomic locus centered on the cis-SNP affects both circulating RSPO3 and trabecular volumetric BMD^37^. Summary statistics for the *RSPO3* locus were available for the *cis*-pQTL identified in the study by Sun et al. but not for the cis-pQTL identified in the study by Emilsson et al.^33^ and consequently the analysis was done only for the *cis*-pQTL identified by Sun et al.^35^.

### Animal experiments

Cell-specific and inducible *Rspo3*-inactivated mouse models were generated by breeding *Rspo3*^*flox/flox*^ mice (*Rspo3*^*tm1.1Jcob*^/J, JAX stock #027313, Jackson Laboratories)^41^ with mice expressing cre recombinase under the control of different promoters. In the *Rspo3*^*flox*^ mice, *LoxP* sites are introduced upstream from *Rspo3* exon 2 and downstream from *Rspo3* exon 4 (Fig. 3a) and in the presence of an active cre recombinase, the DNA fragment including exons 2–4 of the *Rspo3* allele is excised.

All procedures involving animals were approved by the Ethics Committee in Gothenburg, Västra Götaland, and the care of the animals was in compliance with all relevant ethical regulations for animal testing and research. The mice were housed in a standard animal facility with a 12 h dark/light period, at standard temperature and humidity. Food and water were available ad libitum. At termination, the animals were anesthetized with Ketador/Dexdomitor (Richter Pharma/Orion Pharma), bled, and euthanized by cervical dislocation. Long bones and vertebrae were dissected and stored for future analyses, soft tissues were collected, weighed, and snap-frozen in liquid nitrogen.

#### Generation of Runx2-creRspo3^flox/flox^ mice

To generate specific inactivation of *Rspo3* in the osteoblast-lineage, *Rspo3*^*flox/flox*^ mice were bred with *Runx2-cre* mice^10,42^. The *Runx2-creRspo3*^*flox/flox*^ offspring display early osteoblast-specific *cre* expression and have the capacity to recombine *LoxP*-flanked DNA sequences in an early osteoblast-specific manner and recombination occurs at all sites of endochondral and intramembranous bone formation, particularly in periosteal cells, osteoblasts and osteocytes but not osteoclasts, when *Runx2-cre* mice were crossed to a Rosa26 reporter strain^42^. The littermate control mice were *Rspo3*^*flox/flox*^. *Runx2-cre* mice have an unchanged skeletal phenotype compared to WT mice^10^.

#### Generation of Dmp1cre-Rspo3^flox/flox^ mice

To generate mice depleted of *Rspo3* in late osteoblasts and osteocytes, *Rspo3*^*flox/flox*^ female mice were crossed with heterozygous *Rspo3*^+/*flox*^ male mice expressing cre recombinase driven by the 10-kb *Dmp1* promoter specifically expressed in late osteoblasts and osteocytes (*Tg(Dmp1-cre)1Jqfe* mice) (*Dmp1-cre* mice)^43^. The littermate control mice were *Rspo3*^*flox/flox*^. We have previously demonstrated that efficient recombination occurs in osteocytes but not osteoblasts or osteoclasts when *Dmp1-cre* mice were crossed to a Rosa26 reporter strain^68^.

#### Generation of mice with inducible Rspo3 inactivation

In order to study the acute adult effects of *Rspo3*, avoiding confounding developmental effects, inducible *Rspo3* knockout mice were created by breeding the *Rspo3*^*flox/flox*^ mice with the CAGGCre-ER (B6.Cg-Tg(CAG-cre/Esr1*)5Amc/J, JAX stock #004682, Jackson Laboratories) transgenic mice^44,69^. The *CAGGCre-ER-Rspo3*^*flox/flox*^ offspring express a tamoxifen-inducible cre-mediated recombinase. To study the effects of *Rspo3* on the adult bone tissue, a long-term study using adult *CAGGCre-ER-Rspo3*^*flox/flox*^ mice were performed. Male 8-week-old *CAGGCre-ER-Rspo3*^*flox/flox*^ and *Rspo3*^*flox/flox*^ littermate control mice were injected with tamoxifen (50 mg kg^−1^; Sigma-Aldrich) for three consecutive days to activate the cre construct. The mice were terminated 38 days after the first tamoxifen injection at the age of 13.5 weeks.

The different mouse models were genotyped using the primers listed in Supplementary Table 1.

### In situ hybridization

The chromogenic in situ hybridization was performed on vertebra L5 from 5-month-old mice, on 8 G needle biopsies from the posterior iliac crest of human healthy volunteers, as well as proximal femur specimens obtained from coxa valga patients undergoing corrective surgery. The bone specimens were fixated in 4% formaldehyde in phosphate buffered saline, followed by demineralization in 15% EDTA with 0.4% PFA for 3 weeks, and paraffin embedded. All human subjects have given their written informed consent, and the study was conducted according to the World Medical Association Declaration of Helsinki and approved by the Danish National Committee on Biomedical Research Ethics, journal no. S-20110112 and S-20120193. 3.5-µm-thick (human) and 6-µm-thick (mouse) consecutive dewaxed sections were in situ hybridized using a modified protocol of the RNAscope 2.5 HD manual protocol (322300, Advanced Cell Diagnostics, Bio-Techne Ltd., Abingdon, UK)^70^. The following probes were used: mouse R-spondin 3 *(Rspo3*, 402011, target sequence 731–2164), human R-spondin 3 (*RSPO3*, 490581, target sequence 38–1546), mouse Runt-related transcription factor 2 (*Runx2*, 414021, target sequence 3838-4821), mouse collagen 1a1 (*Col1a1*, 319371, target sequence 1686–4669), mouse dentin matrix protein 1 (*Dmp1*, 441171, target sequence 689–1543), and mouse tartrate resistant acid phosphatase 5 (*Trap* (*Acp5*), 465001, target sequence 200–1414). Briefly, after pre-treatment, the sections were hybridized with the probe over night at 40 °C, followed by six steps of amplification according to the manufacturer’s instructions. To further amplify the signal, the DAB staining step was omitted and the signal was amplified with digoxigenin-labeled tyramide signal amplification (NEL 748001KT, Akoya Biosciences, Marlborough, MA, USA), detected by alkaline phosphatase-conjugated anti-digoxigenin Fab fragments (11093274910, Sigma-Aldrich), and visualized with Liquid Permanent Red (K0640, DAKO, Carpinteria, CA, USA). The human sections were subsequently immunostained with mouse anti-TRAP antibodies (Clone ZY-9C5, Zymed), labeled with horseradish peroxidase–conjugated anti-mouse IgG polymers (BrightVision, Immunologic, Duiven, the Netherlands), visualized with Deep Space Black (Biocare Medical, Concord, CA, USA). Finally, both mouse and human sections were counterstained with Mayer’s Hematoxylin (MHS1, Sigma-Aldrich) and mounted with Aquatex (1085620050, Sigma-Aldrich).

### Real-time quantitative PCR

Total mRNA was prepared from mouse cortical bone, vertebral body, brain cortex, flushed bone marrow, liver, lung, gonadal fat, spleen, kidney, thymus, muscle, testis, and heart using TRIzol reagent (15596018; Thermo Fischer Scientific) and/or RNeasy Mini Kit (74106; Qiagen). For preparation of total mRNA from cultured cells, the RNeasy Micro Kit (74004; Qiagen) was used.

The mRNA was reversed transcribed to cDNA (4368814; Thermo Fischer Scientific) and real-time PCR analyses were performed using the StepOnePlus Real-Time PCR System (version 2.3, Thermo Fischer Scientific) and the following Assay-on-Demand primer and probe sets: *R-spondin 3* (*Rspo3)*, Mm01188251_m1; *Sclerostin (Sost)*, Mm00470479_m1; *Dickkopf-1 (Dkk1)*, Mm00438422_m1; *R-spondin 1 (Rspo1)*, Mm00507077_m1; *R-spondin 2 (Rspo2)*, Mm00555790_m1; *R-spondin 4 (Rspo4)*, Mm00615419_m1; *Alkaline phosphatase (Alpl)*, Mm00475834_m1; *Acid phosphatase 5 (Acp5)*, Mm00475698_m1; *Collagen type 1 alpha 1 (Col1a1)*, Mm00801666_g1; *Tumor necrosis factor receptor superfamily, member 11b (Tnfrsf11b, Opg*), Mm00435452_m1; *Tumor necrosis factor superfamily member 11 (Tnfsf11, Rankl*), Mm00441908_m1; *Ki67 (Mki67)*, Mm01278617_m1; *Tcf7 (Tcf1)*, Mm00493445_m1; *Lef1*, Mm01310389; *Znrf3*, Mm01191456_m1; and *Rnf43*, Mm00552558_m1. The expressions of each gene were normalized to 18S ribosomal subunit (4310893E; Thermo Fischer Scientific). The 2^−∆∆Ct^ method was used to calculate the relative gene expression.

### Serum analyses

ELISA RatLaps Kit (AC-06F1, Immunodiagnostic Systems, East Boldon, United Kingdom) were used to measure serum levels of C-terminal type I collagen (CTX) fragments to assess bone resorption. To assess bone formation, serum levels of procollagen type I N-terminal propeptide (P1NP) were measured using a Rat/Mouse EIA Kit (AC-33F1, Immunodiagnostic Systems).

### Single cell RNA sequencing (scRNA seq)

The recently published single cell RNA seq data from *Cxcl12-eGFP* expressing cells was used to study *Rspo3* expression in bone marrow stromal cells^39^. Both data sets (GSM4064136 Cxcl12GFPCxcl12CE#1 and GSM4064137 Cxcl12GFPCxcl12CE#2) was downloaded from GEO project GSE136970 (https://www.ncbi.nlm.nih.gov/geo/query/acc.cgi?acc=GSE136970), imported into R and analyzed using Seurat^39^. Cells with less than 1000 genes per cell were filtered out and the two datasets were merged, resulting in 8026 cells from the two datasets. We further filtered out cells with more than 15 % mitochondrial read content, resulting in 7332 cells remaining for analysis. Further analysis was performed after normalization of the data using the logNormalize method and uniform manifold approxiamtion and projection (UMAP) cluster analysis was done using the first 10 principal components (1:10 dimensions). We obtained 8 different cell clusters (scRNA seq Fig. 1 and scRNA seq Supplementary Fig. 1). Heat map and feature plots of selected genes identified the clusters as four osteogenic/adipogenic (cluster 0, 1, 4 and 7), one mitotic (cluster 6), one endothelial (cluster 5) and two haematopoetic (cluster 2 and 3) (scRNA seq Fig. 1). In contrast to Matsushita et al we kept the haematopoetic clusters in our subsequent *Rspo3* analysis. *Rspo3* expression was in addition analyzed in the nicheExplorer made available by Iannis Aifantis lab (http://aifantislab.com/niche) in an interactive data browser as a supplement to their published scRNAseq data of mouse bone marrow vascular, perivascular, and osteoblast cell populations^40^.

### Assessment of bone parameters

#### Bone histomorphometry

For dynamic histomorphometry, the mice were intraperitoneally injected with the fluorochromes calcein and alizarin (Merck GmbH, Darmstadt, Germany) 9 and 2 days prior to termination, respectively^10^. Upon dissection, vertebra L5 were fixated in 4% formaldehyde, dehydrated in EtOH, and imbedded in methyl methacrylate. Static trabecular bone parameters were determined in a 4-μm-thick plastic sections stained in Masson-Goldner Trichrome and dynamic trabecular bone parameters were determined in unstained 8-μm-thick sections. All parameters were analyzed using the OsteoMeasure7 histomorphometry system (OsteoMetrics, Atlanta, GA, USA), following the guidelines of the American Society for Bone and Mineral Research^71^.

#### High resolution micro-computed tomography

High-resolution micro-computed tomography (µCT) were used to analyze lumbar vertebra 5 (Skyscan, 1172; Bruker MicroCT, Aartselaar, Belgium)^10^. The lumbar vertebra were imaged with an X-ray tube voltage of 50 kV and a current of 201 µA, with a 0.5 mm aluminum filter, and the scanning angular rotation was 180°, and the angular increment was 0.70°. NRecon (version 1.6.9.8, Bruker MicroCT) was used to perform reconstruction after scans. The trabecular bone was analyzed 235 µm from the lower end of the pedicles and continued for approximately 229 µm. The data was analyzed using the CTAn software (version 1.13.2.1, Bruker MicroCT).

#### Peripheral quantitative computed tomography

Computed tomography (pQCT) scans were performed using the peripheral quantitative computed tomography XCT Research M (v.4,5B; Norland Stratec, Pforzheim, Germany), at a resolution of 70 µm^72^. The cortical bone parameters were analyzed in the mid-diaphyseal region of the left femur to determine cortical volumetric bone mineral density (BMD) and the cortical thickness^73^.

#### Mechanical strength

Intact lumbar vertebra 5 was axially loaded with a press head of 2 mm in diameter, with a 1 mm-thick holder through the vertebral foramen, at a speed of 0.155 mm s^−1^ using a mechanical testing machine (Instron 3366, Instron)^74^. The results were calculated by a custom Excel macro using the computer recorded load deformation raw data curves produced by Bluehill 2 software v2.6 (Instron).

### Cell culture media

Primary murine osteoblasts and osteoclasts were cultured in complete α-MEM medium (MEM alpha medium (Gibco, 22561-021) supplemented with 10% heat-inactivated FBS (Sigma, F7524), 2 mM GlutaMAX (Gibco, 35050-038), 50 μg ml^−1^ gentamicin (Gibco, 15750-037), 100 U  ml^−1^ penicillin and 100 μg ml^−1^ streptomycin (Gibco, 15140-148)). For osteoblast differentiation and mineralization assays, mouse calvarial osteoblasts were cultured in complete osteogenic α-MEM supplemented as described above and with 4 mM β-glycerophosphate disodium salt hydrate (BGP; Sigma, G9422) and 0.28 mM l-ascorbic acid 2-phosphate sesquimagnesium salt hydrate (Asc-2P; Sigma, A8960). For osteoclast differentiation assays, bone marrow macrophages were cultured in complete α-MEM supplemented with 30 ng ml^−1^ M-CSF (R&D Systems, 416-ML-050) and 4 ng ml^−1^ RANKL (R&D Systems, 462-TEC-010).

### Murine primary calvarial osteoblast culture

Murine primary calvarial osteoblasts were isolated from 4 to 6 days old mice by sequential collagenase treatments^75^. Cells in collagenase fractions 6-10 were cultured in complete α-MEM in T75 flasks for 4 days prior to re-seeding the cells at 20,000 cells cm^−2^ in 48-well plates. Osteoblasts were cultured in complete osteogenic α-MEM for indicated times with change of half of the culture media volume every 2–3 days. For gene expression analyses, cells were lysed in RNeasy lysis (RLT) buffer with β-mercaptoethanol (Qiagen), followed by RNeasy micro RNA purification (Qiagen), cDNA synthesis and real-time PCR as described above. DNA was isolated using the DNeasy Blood and Tissue kit (Qiagen) following the manufacturer’s instruction for animal cells. For ALP staining, cells were fixed in citrate buffered acetone and stained according to the commercial kit protocol (Sigma, 85L2-1KT). Mineralized nodules were visualized by fixation in 2.5% glutaraldehyde in 70% ethanol for 5 min, followed by three washes in 70% ethanol. For quantification of mineralization, images were taken using a Jenoptik Gryphax camera connected to a Nikon Eclipse 80i microscope and mineralized surface per well was quantified using the Bioquant OSTEO software (version 20.8.6 volume 1993).

### In vitro ablation of *Rspo3* in cultured calvarial osteoblasts

Primary calvarial osteoblasts from *CAGGCre-ER-Rspo3*^*flox/flox*^ and *Rspo3*^*flox/flox*^ control mice were isolated as above and cultured in complete α-MEM for 2–3 days prior to re-seeding in multiwell plates and culture in complete osteogenic media. Cells seeded in 48-well plates were cultured in osteogenic media for 24 h, followed by addition of tamoxifen (Sigma, ReadyMade solution, SML1666) for additional 24 h, before complete media change and continued culture in complete osteogenic media without tamoxifen. 50 or 100 nM tamoxifen was used and resulted in similar results. Osteoblastic differentiation was followed and analyzed as described above with the time of tamoxifen removal designated day 0. PTH (10 nM, Bachem, H-1660) and PGE2 (1 µM, R&D Systems, 2296) was added the day after tamoxifen removal (day 1) and continued until day 6, when cells were harvested for gene expression analysis as described above.

### Murine bone marrow macrophages and osteoclasts cultures

Mouse bone marrow derived macrophages (BMM) were obtained from 10 to 12 weeks old male wild type C57Bl/6N mice by culture of bone marrow cells in suspension culture dishes in complete α-MEM supplemented with 30 ng ml^−1^ M-CSF (R&D Systems, 416-;L-050)^76,77^. BMMs were spot seeded in 48-well plates (20,000 cells in 20 μl complete α-MEM) and allowed to attach for 5 min before addition of complete α-MEM supplemented with 30 ng ml^−1^ M-CSF, with or without 4 ng ml^−1^ RANKL (R&D Systems, 462-TEC-010) and 100 ng ml^−1^ recombinant RSPO3 (R&D Systems, 4120-RS-025) for 3 days to induce osteoclast differentiation. Cells were stained for tartrate resistant acid phosphatase (TRAP) after 3 days using a commercial kit (Sigma, 387A-KT1). TRAP-positive cells with at least three nuclei were defined as osteoclasts and counted using the Bioquant OSTEO software. For gene expression analyses, cells were lysed in RLT buffer with β-mercaptoethanol (Qiagen), followed by RNeasy micro RNA purification (Qiagen), cDNA synthesis and real-time PCR as described above.

### Protein preparation and analyses

Primary calvarial osteoblasts from *CAGGCre-ER-Rspo3*^*flox/flox*^ and *Rspo3*^*flox/flox*^ control mice were isolated as above and cultured in complete α-MEM for 2–3 days prior to re-seeding in 12-well plates at 20,000 cells cm^−2^. Cells were then cultured in osteogenic media for 24 h, after which 100 nM tamoxifen was added for 24 h, followed by complete media change to osteogenic media without tamoxifen. Protein lysates were prepared 24 h after removal of tamoxifen by washing the cells once with cold PBS followed by addition and lysis in RIPA buffer (Sigma, R0278) with protease inhibitors (complete Mini EDTA-free; RocheDiagnostics, 5892970001) and phosphatase inhibitors (PhosSTOP; Roche Diagnostics, 04906845001). Lysates were transferred to tubes, centrifuged for 10 min at 11,000×*g*, +4 °C, and the supernatant was used for protein analysis. Protein concentration was quantified using the DC Protein Assay Kit (Bio-Rad, 500-0112). Levels of LRP6 and pLRP6 were analyzed by capillary-based electrophoresis and immunodetection using the JESS ProteinSimple system and the Compass for SW Software (version 5.0.0, Protein Simple) as described by the manufacturer. Protein lysates were analyzed with anti-LRP6 (clone C47E12, Cell Signaling Technology, 3395, dilution 1:250) or anti-pLRP6 (Ser1490, Cell Signaling Technology, 2568, dilution 1:10) together with the Protein Normalization Assay Module (ProteinSimple, AM-PN01) and the Anti-rabbit Detection Module (ProteinSimple, DM-001) on 12–230 kDa capillary separation cassettes (ProteinSimple, SM-W004). LRP6 and pLRP6 are visually presented in the lane view of the Compass Software and quantified by normalization to total protein levels according to the SimpleWestern protocol and following the guidelines for presentation and quantification of protein data by Journal of Biological Chemistry^78^.

### Reporting summary

Further information on research design is available in the Nature Research Reporting Summary linked to this article.

## Supplementary information

Supplementary Information

Reporting Summary

## Data Availability

For the human association studies we have used data from UK Biobank. Access to the UKB Resource is available by application (http://www.ukbiobank.ac.uk/). Access to data from the UK Biobank can be obtained at https://www.ukbiobank.ac.uk/enable-your-research. The UK Biobank resource is available to bona fide researchers for health-related research in the public interest. All researchers who wish to access the research resource must register with UK Biobank by completing the registration form in the Access Management System. Registrations are according to UK Biobank reviewed within 10 working days of submission (https://www.ukbiobank.ac.uk/enable-your-research/register). After registration is approved, applications on access to the UK Biobank research resource can be submitted. Research applications to access the data are performed via the Access Management System (https://bbams.ndph.ox.ac.uk/ams/). The datasets used for the look ups of fracture signals at the *RSPO3* locus are detailed in the “Methods” section, and have been published by Morris et al.^8^, GWAS summary statistics are available at the GEFOS website http://www.gefos.org, associations with eQTLs for the three SNPs at the RSPO3 locus were evaluated using the GTEx Portal https://gtexportal.org/home/, and the associations for the cis-pQTLs for RSPO3 were taken from two independent datasets, including rs3734626 from Emilsson et al.^33^ and rs2489623 from Sun et al.^35^. Single cell RNA sequencing (scRNA seq) data: scRNA seq data have previously been deposited in GEO, accession number GSE136970^39^ and GSE108892^40^. Source data are provided with this paper.

## Source data

Source Data
