# Supplementary material for: RSPO3 is important for trabecular bone and fracture risk in mice and humans
Source: Nat Commun. 2021 Aug 13;12:4923. doi: 10.1038/s41467-021-25124-2 (PMC8363747; doi:10.1038/s41467-021-25124-2)
Supplement: Supplementary file 1 — Supplementary Information [file 41467_2021_25124_MOESM1_ESM.pdf]

Supplementary material

RSPO3 is important for trabecular bone and fracture risk in mice and humans

Karin H. Nilsson[1#], Petra Henning[1#], Maha El Shahawy[1,2], Maria Nethander[1], Thomas L. Andersen[3,4], Charlotte Ejersted[5], Jianyao Wu[1], Karin L. Gustafsson[1], Antti Koskela[6], Juha Tuukkanen[6], Pedro P. C. Souza[7], Jan Tuckermann[8], Mattias Lorentzon[1,9,10], Linda Engström Ruud[11], Terho Lehtimäki[12,13], Jon H Tobias[14], Sirui Zhou[15,16], Ulf H. Lerner[1#], J Brent Richards[15,16#], Sofia Movérare-Skrtic[1*#] and Claes Ohlsson[1,17*#]

[1]Sahlgrenska Osteoporosis Centre, Centre for Bone and Arthritis Research, Institute of Medicine, Sahlgrenska Academy at University of Gothenburg, Gothenburg, Sweden

[2]Present address: Faculty of Dentistry, Department of Oral Biology, Minia University, Minia 61511, Egypt

[3]Department of Clinical Research, University of Southern Denmark, Odense, Denmark

[4]Department of Pathology, Odense University Hospital, Odense, Denmark

[5]Department of Endocrinology, Odense University Hospital, Odense, Denmark

[6]Department of Anatomy and Cell Biology, Faculty of Medicine, Institute of Cancer Research and Translational Medicine, University of Oulu, Oulu, Finland

[7]Innovation in Biomaterials Laboratory, Faculty of Dentistry, Federal University of Goiás, Goiâna, Brazil

[8]Institute of Comparative Molecular Endocrinology (CME), University of Ulm, Ulm, Germany

[9]Region Västra Götaland, Department of Geriatric Medicine, Sahlgrenska University Hospital, Mölndal, Sweden

[10] Mary MacKillop Institute for Health Research, Australian Catholic University, Melbourne, VIC, Australia

[11]Department of Physiology, Institute of Neuroscience and Physiology, The Sahlgrenska Academy at the University of Gothenburg, Gothenburg, Sweden

[12]Department of Clinical Chemistry, Fimlab Laboratories

[13]Finnish Cardiovascular Research Center – Tampere, Faculty of Medicine and Health Technology, Tampere University, Tampere 33520, Finland

[14]Musculoskeletal Research Unit, Translational Health Sciences, and Medical Research Council Integrative Epidemiology Unit, Bristol Medical School, University of Bristol, Bristol, UK

[15]Centre for Clinical Epidemiology, Department of Medicine, Lady Davis Institute, Jewish General Hospital, McGill University, Montréal, Québec, Canada

[16]Department of Human Genetics, McGill University, Montréal, Québec, Canada

[17]Region Västra Götaland, Department of Drug Treatment, Sahlgrenska University Hospital, Gothenburg, Sweden

[#]Contributed equally

* Corresponding author:

Centre for Bone and Arthritis Research, Vita Stråket 11, Sahlgrenska University Hospital, S-413 45 Gothenburg, Sweden. E-mail: claes.ohlsson@medic.gu.se and sofia.skrtic@gu.se

Supplementary figure 1

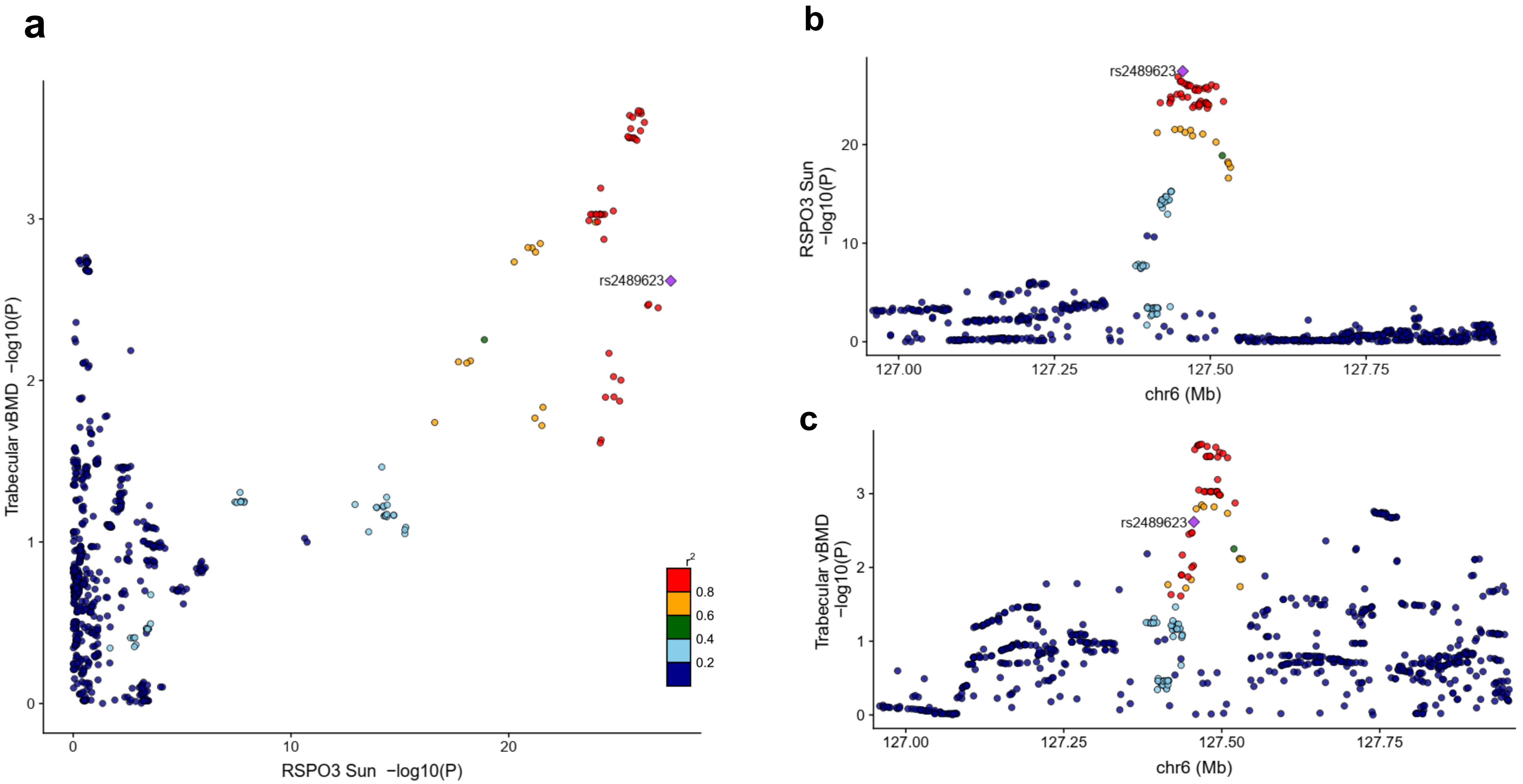

Supplementary Figure 1:. Genetic colocalization of circulating RSPO3 and trabecular volumetric BMD

(a) Colocalization of circulating RSPO3 from Sun *et al* (x-axis)[1] and trabecular volumetric bone mineral density (vBMD; y-axis)[2] GWAS, showing the correlation of P values of SNPs within 1MB regions around the cis-SNP of circulating RSPO3 (highlighted in purple diamond) and trabecular volumetric BMD GWAS. Created by LocusCompare R package. Bayesian colocalization analyses using COLOC[3] found that the plasma RSPO3 signal identified by Sun *et al* was colocalized with the signal observed for trabecular vBMD in the *RSPO3* locus with a posterior probability of shared signal of 72% (PP.H4 = 0.72), indicating that there is a single shared genetic signal in the 1MB locus of the *RSPO3* cis-pQTL SNP (rs2489623) affecting both circulating RSPO3 and trabecular vBMD.

(b-c) Locuszoom plots at the *RSPO3* locus, showing the regional association in circulating RSPO3 GWAS (b) and trabecular vBMD GWAS (c). Purple diamond indicates the circulating RSPO3 cis-SNP (rs2489623) based on the Sun *et al* GWAS.

Supplementary figure 2

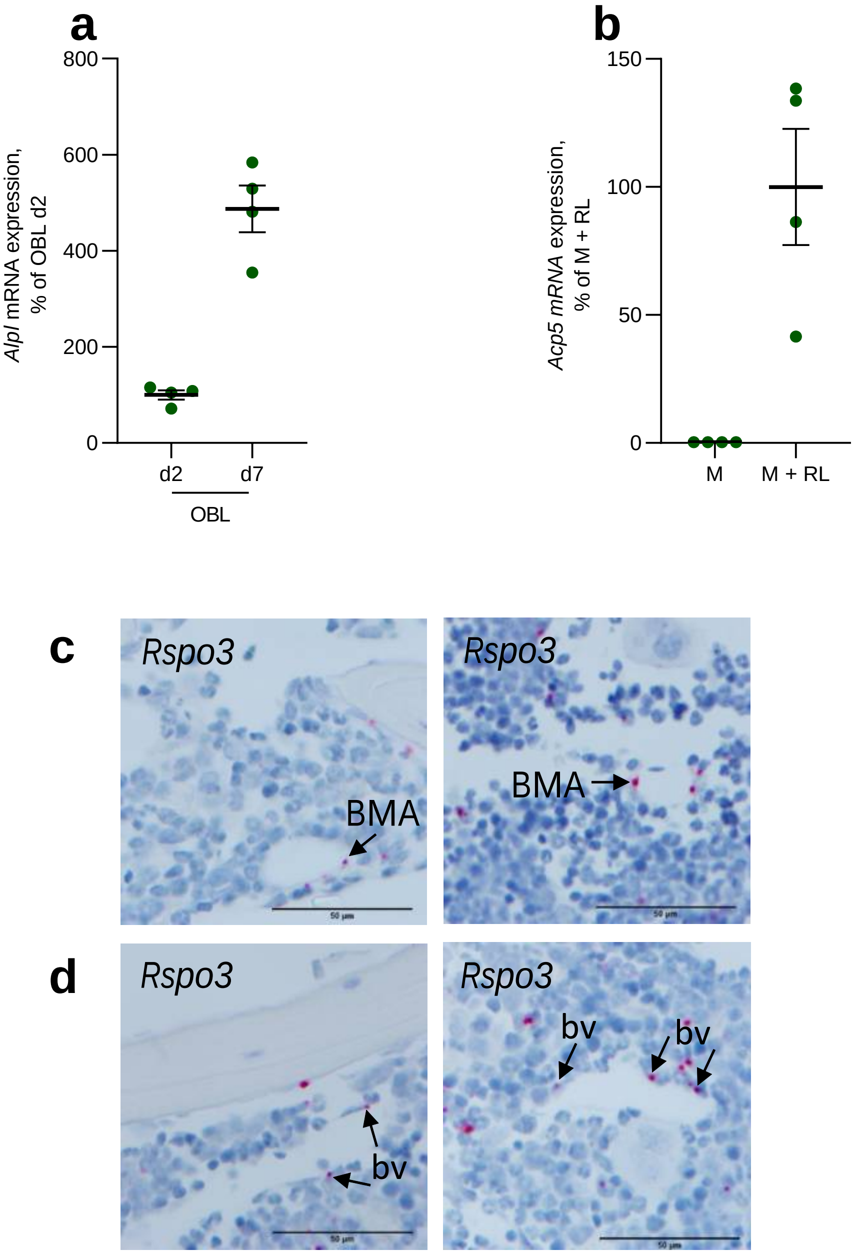

Supplementary Figure 2: Differentiation of osteoblasts and osteoclasts, and the expression of RSPO3 in the bone marrow

(a) Expression of *Alpl* mRNA in primary calvarial osteoblast cultured for 2 and 7 days in osteogenic media (for the cells presented in Fig 1b). Individual values are presented in all graphs with the mean presented as horizontal lines and ± SEM as vertical lines. (n=4 wells per group).

(b) Expression of *Acp5* mRNA in bone marrow macrophages (BMM) and RANKL-stimulated osteoclasts (OCL; for the cells presented in Fig 1b). Individual values are presented in all graphs with the mean presented as horizontal lines and ± SEM as vertical lines. (n=4 wells per group).

(c, d) Representative *in situ* hybridization data in transverse sections across lumbar vertebra 5 in wildtype mouse showing the mRNA expression pattern (red) of *Rspo3* in bone marrow adipocytes (BMA; c) and in a subset of cells close to blood vessels (BV; d). Experiments were repeated at least three times. Source data are provided as a Source Data file.

Supplementary figure 3

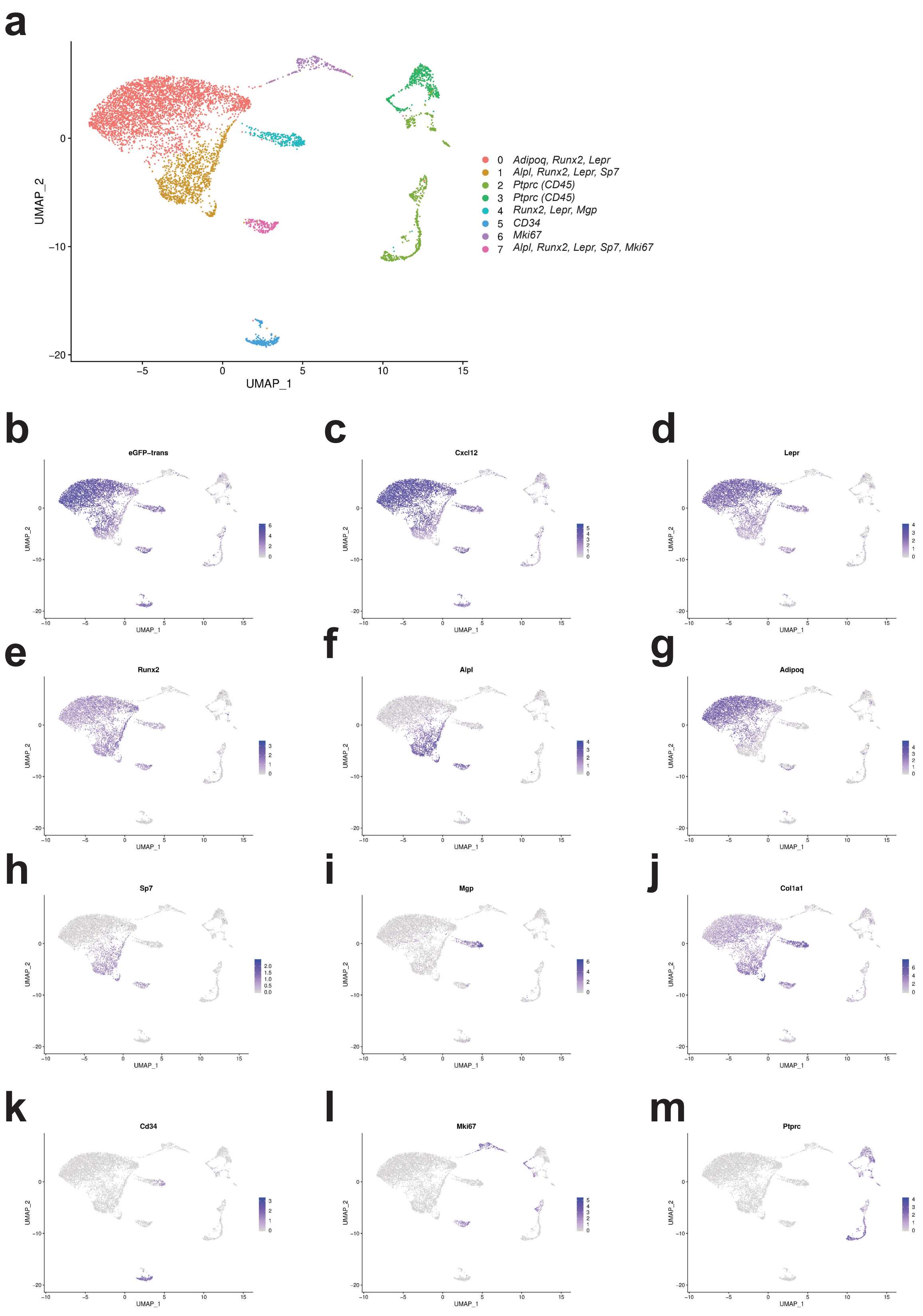

Supplementary Figure 3: *Rspo3* expression in *Cxcl12* expressing bone marrow stromal cells

Single cell RNA sequencing analysis of *Cxcl12* expressing bone marrow cells[4]. (a) UMAP-based visualization of major cell clusters (Cluster 0-7). (b-m) Feature plots of selected cluster expressed genes; sGFP-trans (b), *Cxcl2* (c), *Lepr* (d), *Runx2* (e), *Alpl* (f), *Adipoq* (g), *Sp7* (h), *Mgp* (i), *Col1a1* (j), *Cd34* (k), *Mki67* (l), and *Pfprc* (m).

Supplementary figure 4

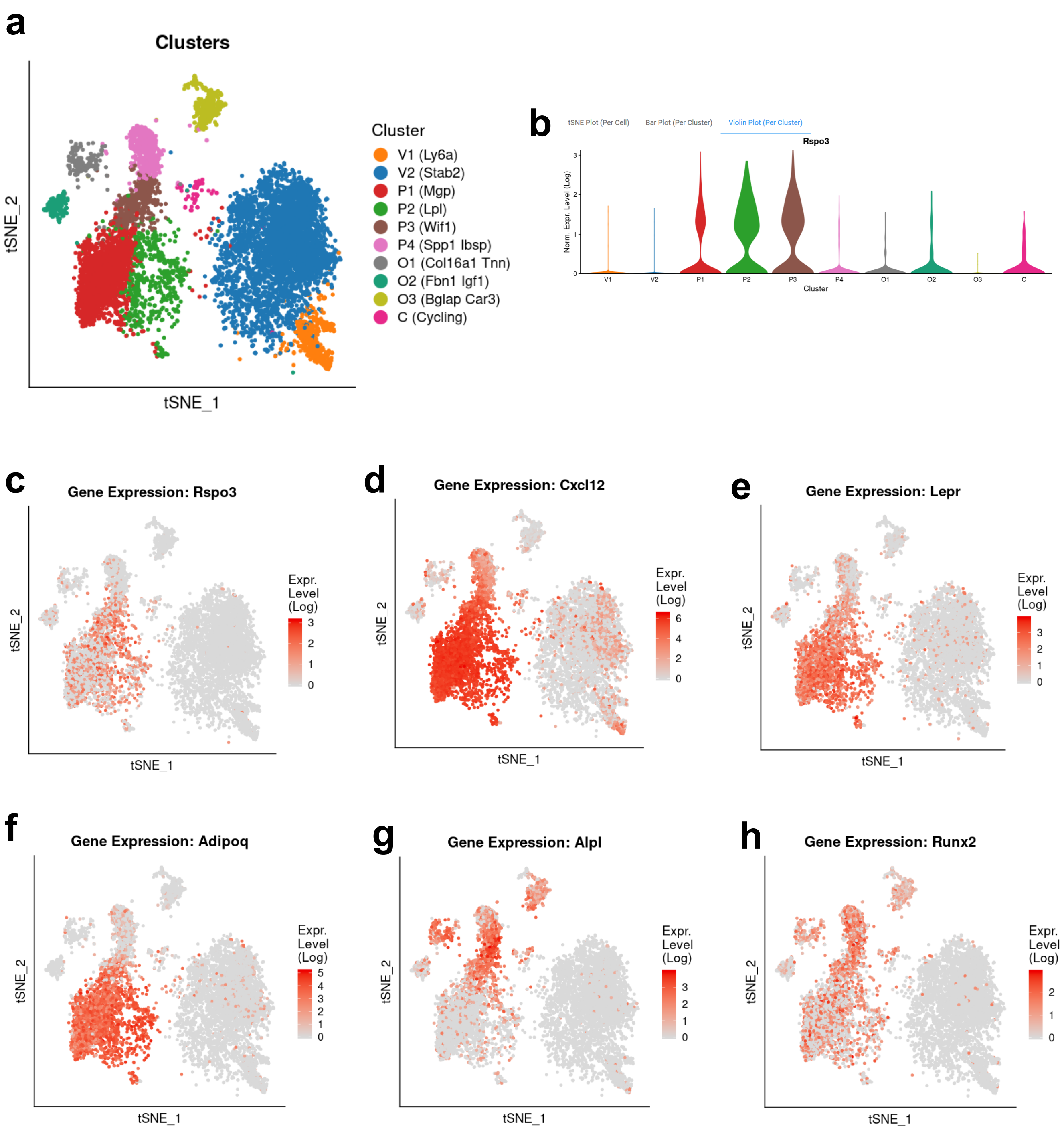

Reprinted with permission from **Springer Nature Costumer Service Centre GmbH; Springer Nature; Nature;** The bone marrow microenvironment at single-cell resolution, Anastasia N. Tikhonova et al., **©2019,**
www.aifantislab.com/niche

Supplementary Figure 4: *Rspo3* expression in *Cdh5* (found in vasculature), *Lepr* (perivascular stromal stem cells) and *Col1a1* (osteoblastic cells) expressing bone marrow cells

Single cell RNA sequencing analysis using nicheExplorer[5]. (a) *t*-SNE visualization of two endothelial (V1-2), four perivascular (P1-4), three osteo-lineage (O1-3) and one mitotic cell cluster identified by Tikhovona et al[5]. Violin plot (b), and feature plot (c) of *Rspo3* expression. Feature plots of *Cxcl12* (d), *Lepr* (e), *Adipoq* (f), *Alpl* (g) and *Runx2* (h) expression.

Reprinted with permission from **Springer Nature Costumer Service Centre GmbH; Springer Nature; Nature;** The bone marrow microenvironment at single-cell resolution, Anastasia N. Tikhonova et al., **©2019,** www.aifantislab.com/niche

Supplementary figure 5

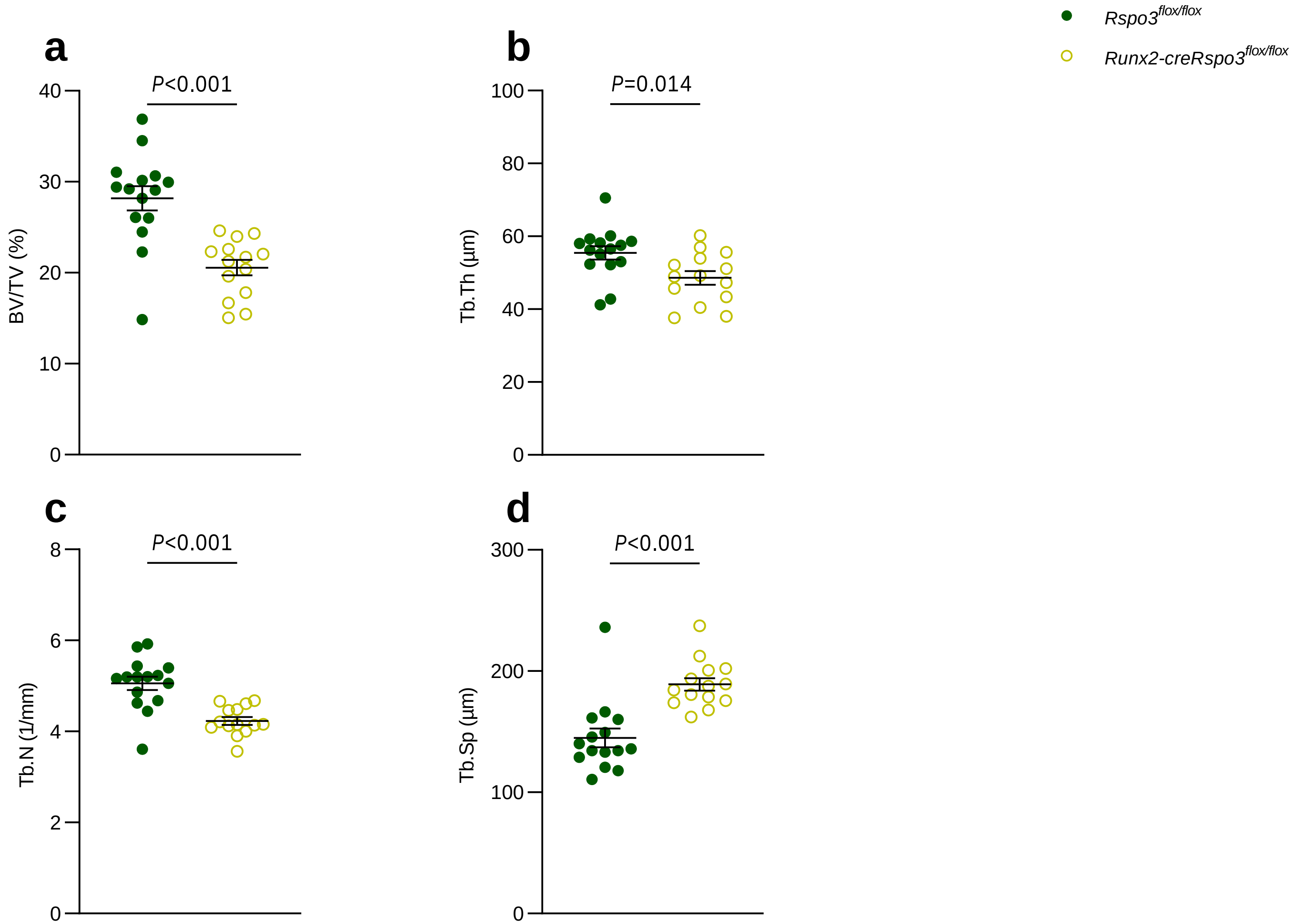

e

	Rspo3$^{flox/flox}$	*Runx2-creRspo3*$^{flox/flox}$
Static histomorphometry		
The number of osteoclasts per bone perimeter (N.Ocl/B.Pm; mm^{-1})	0.91 ± 0.11	1.11 ± 0.12
The osteoclast surface per trabecular bone surface (Ocl.S/BS; %)	2.48 ± 0.30	2.84 ± 0.26
The number of osteoblasts per bone perimeter (N.Obl/B.Pm; mm^{-1})	3.34 ± 0.23	3.67 ± 0.46
Osteoblast surface per trabecular bone surface (Obl.S/BS, %)	4.80 ± 0.37	5.42 ± 0.75
Dynamic histomorphometry		
Mineralizing surface per bone surface (MS/BS; %)	35.8 ± 1.65	37.7 ± 2.15
Mineral apposition rate (MAR; µm/d)	1.56 ± 0.04	1.60 ± 0.06
Bone formation rate per tissue volume (BFR/TV; %/y)	207 ± 12.8	175 ± 13.9
Bone formation rate per bone surface (BFR/BS, µm^3/µm^2/y)	203 ± 11.2	222 ± 16.8

Values are given as mean ± standard error of the mean. (*Runx2-creRspo3*$^{flox/flox}$, n=14; *Rspo3*$^{flox/flox}$, n=15).

f

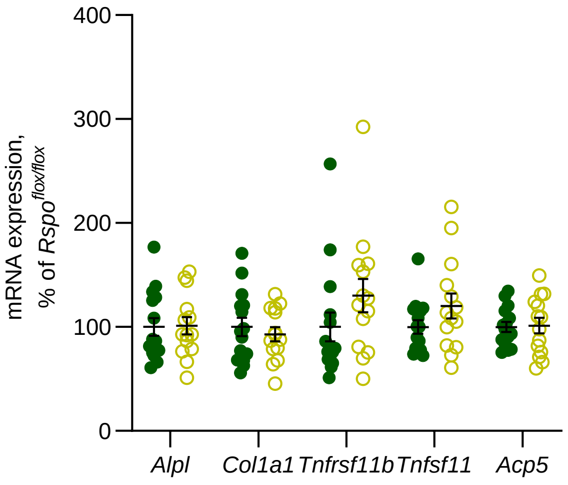

Supplementary Figure 5: Histomorphometry revealed reduced trabecular bone volume fraction in *Runx2-creRspo3$^{flox/flox}$* mice

(a-d) Trabecular bone volume over tissue volume (BV/TV; a), trabecular thickness (Tb,Th; b), trabecular number (Tb.N; c), and trabecular separation (Tb.Sp; d) of vertebra L5 in 13-week-old male *Runx2-creRspo3$^{flox/flox}$* (n=14), compared to *Rspo3$^{flox/flox}$* (n=15), as measured by bone histomorphometry.

(e) Table of static and dynamic histomorphometry of trabecular vertebrae L5 in 13-week-old male *Runx2-creRspo3$^{flox/flox}$* (n=14), compared to *Rspo3$^{flox/flox}$* (n=15).

(f) mRNA expression of *Alpl, Col1a1, Tnfrsf11b, Tnfsf11,* and *Acp5* in vertebral body in 13-week-old male *Runx2-creRspo3$^{flox/flox}$* (n=14) mice, compared to *Rspo3$^{flox/flox}$* (n=15) mice.

Individual values are presented in all graphs with the mean presented as horizontal lines and ± SEM as vertical lines. Statistics were analysed using two-sided Student's *t* test. Source data are provided as a Source Data file.

Supplementary figure 6

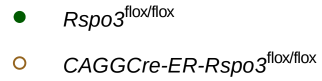

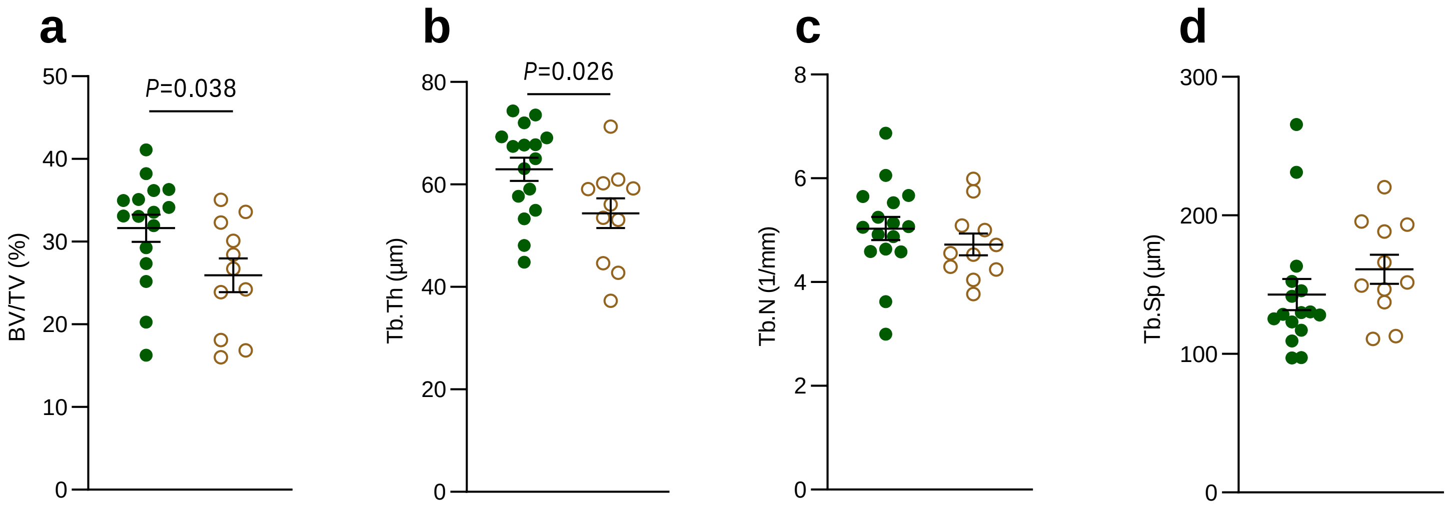

e

	Rspo3$^{flox/flox}$	*CAGGCre-ER-Rspo3$^{flox/flox}$*
Mineralizing surface per trabecular bone surface (MS/BS; %)	39.2 ± 1.83	35.8 ± 2.02
Mineral apposition rate (MAR; mm/d)	1.61 ± 0.08	1.48 ± 0.12
Bone formation rate per tissue volume (BFR/TV; %/y)	257 ± 27.4	178 ± 20.5*
Bone formation rate per trabecular bone surface (BFR/BS; mm^3/mm^2/y)	235 ± 20.8	194 ± 17.7

Values are given as mean ± standard error of the mean. (*CAGGCre-ER-Rspo3$^{flox/flox}$*, n=9; *Rspo3$^{flox/flox}$*, n=17). **P* < 0.05 vs *Rspo3$^{flox/flox}$* using Student's *t* test in male 13-week-old mice.

g

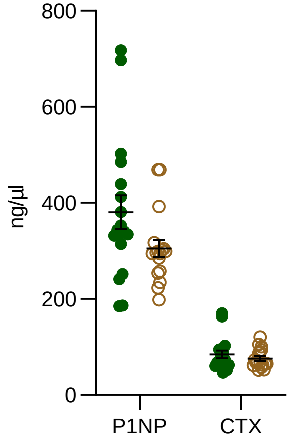

f

	Rspo3$^{flox/flox}$	*CAGGCre-ER-Rspo3$^{flox/flox}$*
The number of osteoblasts per trabecular bone perimeter (N.Obl/B.Pm; mm^{-1})	5.14 ± 0.70	3.51 ± 0.43
Osteoblast surface per trabecular bone surface (Obl.S/BS; %)	9.16 ± 1.52	5.93 ± 0.98
The number of osteoclasts per bone perimeter (N.Ocl/B.Pm; mm^{-1})	0.97 ± 0.10	1.14 ± 0.12
The osteoclast surface per trabecular bone surface (Ocl.S/BS; %)	2.97 ± 0.30	3.22 ± 0.37

Values are given as mean ± standard error of the mean. (*CAGGCre-ER-Rspo3$^{flox/flox}$*, n=9; *Rspo3$^{flox/flox}$*, n=17).

Supplementary Figure 6: Histomorphometry revealed reduced trabecular bone volume fraction in *CAGGCre-ER-Rspo3$^{flox/flox}$* mice

(a-d) Trabecular bone volume over tissue volume (BV/TV; a), trabecular thickness (Tb.Th; b), trabecular number (Tb.N; c), and trabecular separation (Tb.Sp; d), in vertebra L5 in male 13-week-old male *CAGGCre-ER-Rspo3$^{flox/flox}$* (n=11) mice, compared to *Rspo3$^{flox/flox}$* (n=16) mice, as measure by bone histomorphometry.

(e) Table of dynamic trabecular bone parameters in vertebra L5 in male 13-week-old male *CAGGCre-ER-Rspo3$^{flox/flox}$* (n=9) mice, compared to *Rspo3$^{flox/flox}$* (n=13) mice, as measured by bone histomorphometry.**P*=0.047

(f) Table of static trabecular bone parameters in vertebra L5 in male 13-week-old male *CAGGCre-ER-Rspo3$^{flox/flox}$* (n=11) mice, compared to *Rspo3$^{flox/flox}$* (n=16) mice, as measured by bone histomorphometry.

(g) Levels of procollagen type I N-terminal propeptide (P1NP, left) and C-terminal type I collagen (CTX, right) in 13-week-old male *CAGGCre-ER-Rspo3$^{flox/flox}$* (n=11) mice, compared to *Rspo3$^{flox/flox}$* (n=17) mice.

Individual values are presented in all graphs with the mean presented as horizontal lines and ± SEM as vertical lines. Statistics were analysed using two-sided Student's *t* test. Source data are provided as a Source Data file.

Supplementary figure 7

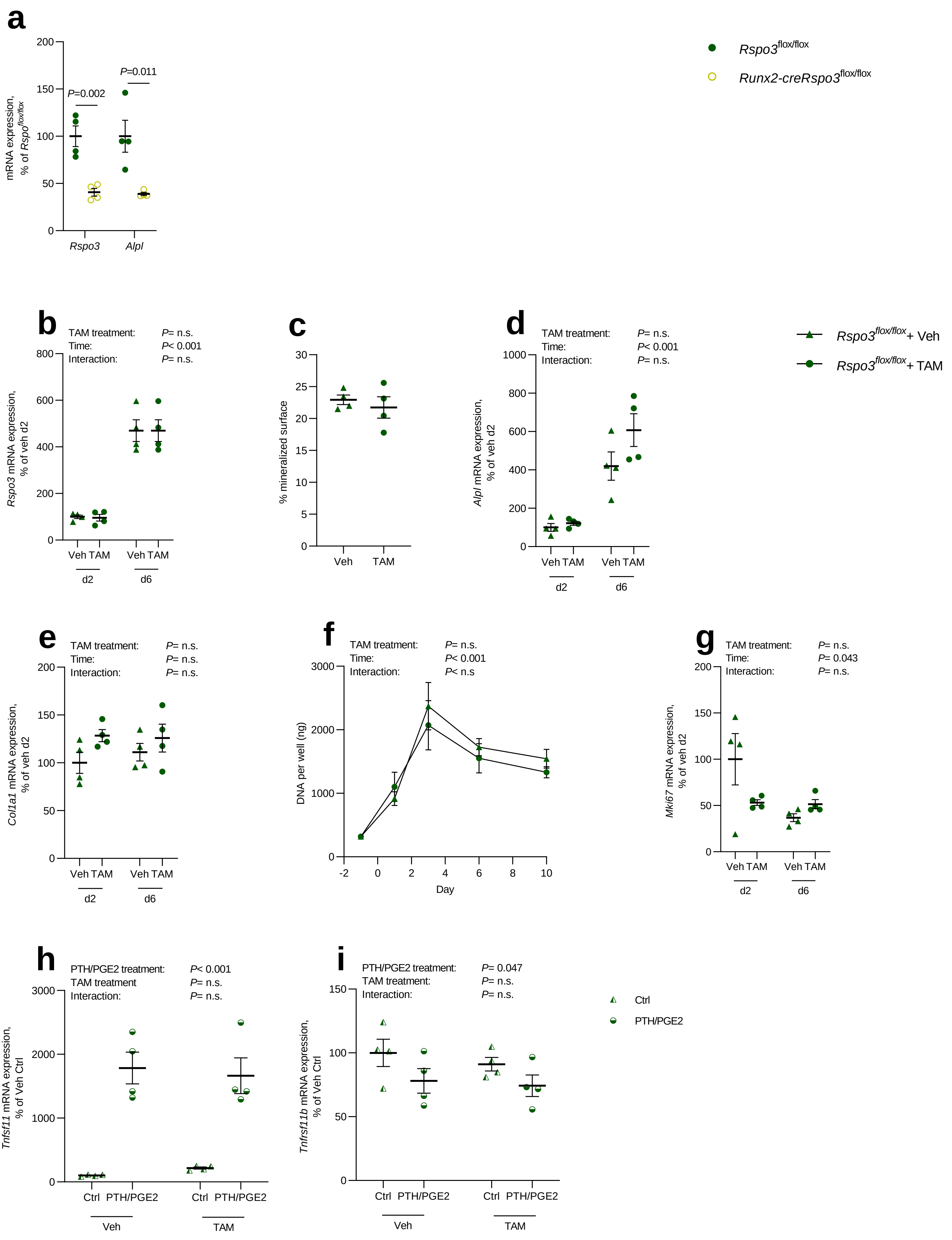

Supplementary Figure 7: The effect of RSPO3 on osteoblast proliferation and differentiation in primary calvarial osteoblasts

(a) mRNA expression analysis of *Rspo3* and *Alpl* in primary calvarial osteoblasts isolated from *Runx2-creRspo3*$^{flox/flox}$ mice cultured in osteogenic media for 3 days. (n=4 wells per group).

(b) mRNA expression analysis of *Rspo3* in primary calvarial osteoblasts isolated from *Rspo3*$^{flox/flox}$ mice cultured in osteogenic media for 2 and 6 days with or without prior *Rspo3* inactivation using tamoxifen (TAM). (n=4 wells per group)

(c) Quantification of mineralized surface per well in *Rspo3*$^{flox/flox}$ cells in fig 5c. (n=4 well per group)

(d, e) mRNA expression analysis of *Alpl* (d) and *Col1a1* (e) in primary calvarial osteoblasts isolated from *Rspo3*$^{flox/flox}$ mice cultured in osteogenic media for 2 and 6 days with or without prior *Rspo3* inactivation using TAM. (n=4 wells per group)

(f) Amount of DNA per well in primary *Rspo3*$^{flox/flox}$ calvarial osteoblasts directly after removal of TAM (day 0, d0) and after culture in osteogenic media for 2 days (d2). (n=4 wells per group)

(g) mRNA expression analysis of *Mki67* in primary *Rspo3*$^{flox/flox}$ calvarial osteoblasts cultured in osteogenic media for 2 days after *Rspo3* inactivation using TAM. (n=4 wells per group).

(h, i) mRNA expression analysis of *Tnfsf11* (h) and *Tnfrsf11b* (i) in primary calvarial osteoblasts isolated from *CAGGCre-ER-Rspo3*$^{flox/flox}$ mice cultured in osteogenic media with or without PTH and PGE2 for 5 days with or without prior *Rspo3* inactivation using TAM. (n=4 wells per group).

Individual values are presented in all graphs with the mean presented as horizontal lines and ± SEM as vertical lines. Two-sided Student's *t* test was used when comparing only two groups (n=4 wells per group). Experiments were repeated two (h-i) or at least three (a-g) times. A two-way ANOVA was used to determine the overall effect of RSPO3 inactivation (treatment with TAM or veh), PTH/PGE2 treatment, time, as well as their interaction. A difference was considered significant when $P<0.05$. n.s. = non-significant Source data are provided as a Source Data file.

Supplementary figure 8

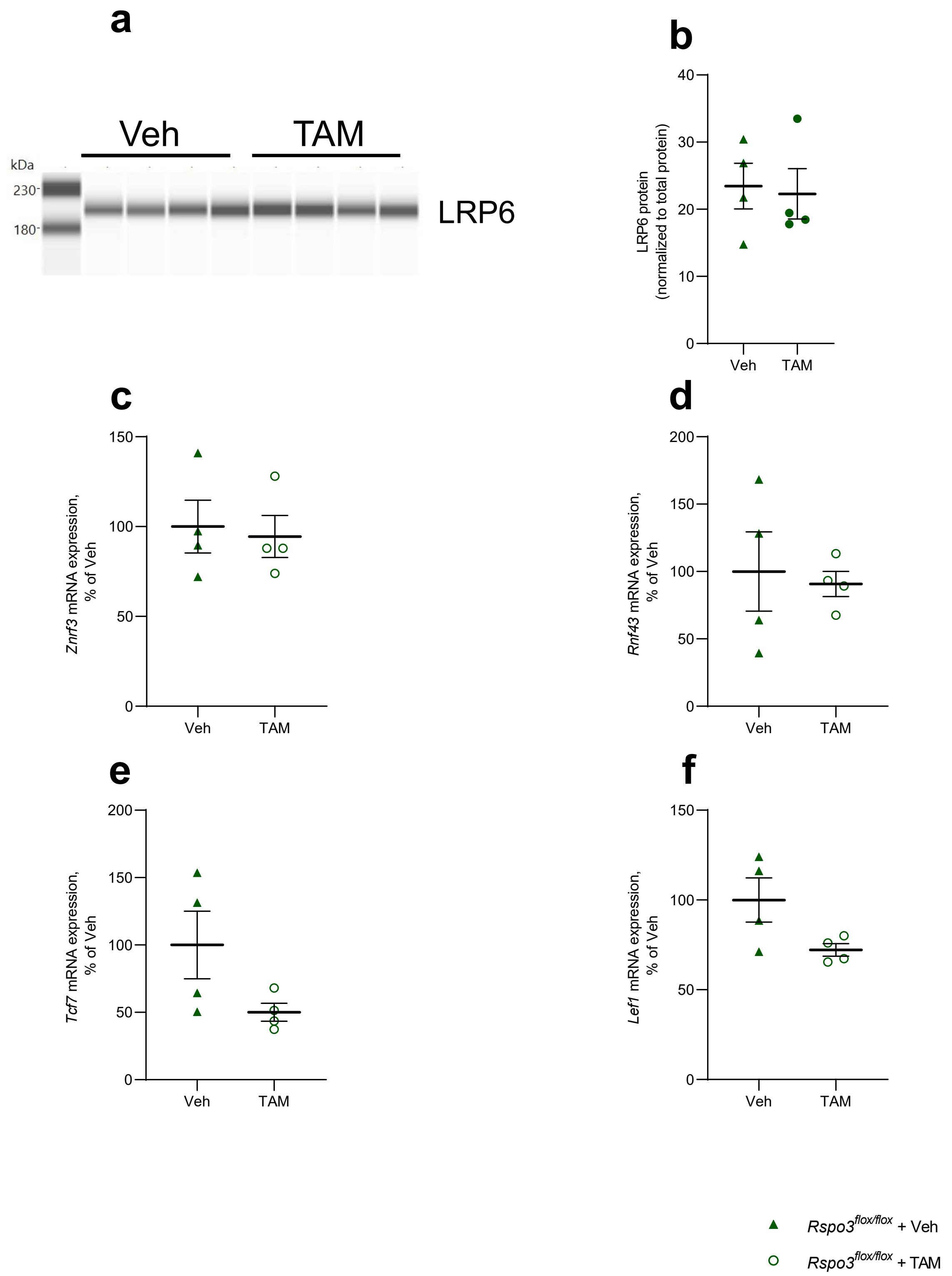

Supplementary Figure 8: The effect of RSPO3 on WNT canonical signalling in primary calvarial osteoblasts

(a, b) LRP6 protein analysis in primary calvarial osteoblasts isolated from *Rspo3*$^{flox/flox}$ mice cultured in osteogenic media for 1 day with or without prior tamoxifen (TAM) treatment as for *Rspo3* inactivation. Lane view from the software in the capillary-based electrophoresis immunodetection system (a) and protein levels (b) normalized to total protein.

(c-f) mRNA expression analyses of *Znrf3* (c), *Rnf43* (d), *Tcf7* (e) and *Lef1* (f) in primary calvarial osteoblasts isolated from *Rspo3*$^{flox/flox}$ mice cultured in osteogenic media for 6 days with or without prior TAM treatment as for *Rspo3* inactivation.

Veh = vehicle. Individual values are presented in all graphs with the mean presented as horizontal lines and ± SEM as vertical lines. Statistics were analysed using two-sided Student's *t* test. n=4 wells per group. Experiments were repeated one (a-d) or two (e-f) times. Source data are provided as a Source Data file.

Supplementary table 1

Primer name	Primer sequence
Rspo3 FP	TAA TGC CCA GGA ACT TTT GG
Rspo3 RP	GCC TAG AAC AGC AAC ATG GAG
cbfa_24	CCA GGA AGA CTG CAA GAA GG
cbaf_25	TTG CTT GCA GGT ACA GGA G
cbfa_30	GGA GCT GCC GAG TCA ATA AC
Dmp1 Cre F	GTT CGC AAG AAC CTG ATG GAC A
Dmp1 Cre R	CTA GAG CCT GTT TTG CAC GTT C
TAM Fwd	GCG GTC TGG CAG TAA AAA CTA TC
TAM rev	GTG AAA CAG CAT TGC TGT CAC TT
TAM WT fwd	CTA GGC CAC AGA ATT GAA AGA TCT
TAM WT rev	GTA GGT GGA AAT TCT AGC ATC ATC C

Primers used for genotyping the animal models used.

Supplementary Table 1: Primers used for genotyping

Primers and primer sequences used for genotyping the animal models.

References

1. Sun, B.B., *et al.* Genomic atlas of the human plasma proteome. *Nature* **558**, 73-79 (2018).
2. Paternoster, L., *et al.* Genetic determinants of trabecular and cortical volumetric bone mineral densities and bone microstructure. *PLoS genetics* **9**, e1003247 (2013).
3. Giambartolomei, C., *et al.* Bayesian test for colocalisation between pairs of genetic association studies using summary statistics. *PLoS genetics* **10**, e1004383 (2014).
4. Matsushita, Y., *et al.* A Wnt-mediated transformation of the bone marrow stromal cell identity orchestrates skeletal regeneration. *Nature communications* **11**, 332 (2020).
5. Tikhonova, A.N., *et al.* The bone marrow microenvironment at single-cell resolution. *Nature* **569**, 222-228 (2019).
