# Supplementary material for: RSPO3 is important for trabecular bone and fracture risk in mice and humans
Source: Nat Commun. 2021 Aug 13;12:4923. doi: 10.1038/s41467-021-25124-2 (PMC8363747; doi:10.1038/s41467-021-25124-2)
Supplement: Supplementary file 2 — Reporting Summary [file 41467_2021_25124_MOESM2_ESM.pdf]

nature portfolio

Corresponding author(s): Sofia Movérare-Skrtic and Claes Ohlsson

Last updated by author(s): Jul 13, 2021

# Reporting Summary

Nature Portfolio wishes to improve the reproducibility of the work that we publish. This form provides structure for consistency and transparency in reporting. For further information on Nature Portfolio policies, see our Editorial Policies and the Editorial Policy Checklist.

## Statistics

For all statistical analyses, confirm that the following items are present in the figure legend, table legend, main text, or Methods section.

| n/a | Confirmed | |
|---|---|---|
| ☐ | ☒ | The exact sample size (*n*) for each experimental group/condition, given as a discrete number and unit of measurement |
| ☐ | ☒ | A statement on whether measurements were taken from distinct samples or whether the same sample was measured repeatedly |
| ☐ | ☒ | The statistical test(s) used AND whether they are one- or two-sided<br>*Only common tests should be described solely by name; describe more complex techniques in the Methods section.* |
| ☐ | ☒ | A description of all covariates tested |
| ☐ | ☒ | A description of any assumptions or corrections, such as tests of normality and adjustment for multiple comparisons |
| ☐ | ☒ | A full description of the statistical parameters including central tendency (e.g. means) or other basic estimates (e.g. regression coefficient) AND variation (e.g. standard deviation) or associated estimates of uncertainty (e.g. confidence intervals) |
| ☐ | ☒ | For null hypothesis testing, the test statistic (e.g. $F$, $t$, $r$) with confidence intervals, effect sizes, degrees of freedom and $P$ value noted<br>*Give P values as exact values whenever suitable.* |
| ☒ | ☐ | For Bayesian analysis, information on the choice of priors and Markov chain Monte Carlo settings |
| ☒ | ☐ | For hierarchical and complex designs, identification of the appropriate level for tests and full reporting of outcomes |
| ☒ | ☐ | Estimates of effect sizes (e.g. Cohen's *d*, Pearson's *r*), indicating how they were calculated |

*Our web collection on statistics for biologists contains articles on many of the points above.*

## Software and code

Policy information about availability of computer code

| | |
|---|---|
| Data collection | NRecon 1.6.9.8<br>CTan 1.13.2.1<br>Compass for SW 5.0.0<br>Bioquant Osteo 20.8.6 volume 1993<br>StepOnePlus v2.3<br>Osteomeasure 7<br>Bluehill software v2.6 |
| Data analysis | GraphPad Prism 9.1.2<br>R 3.5.1<br>Seurat 3.1.2<br>UWOT (for UMAP) O.1.5 |

For manuscripts utilizing custom algorithms or software that are central to the research but not yet described in published literature, software must be made available to editors and reviewers. We strongly encourage code deposition in a community repository (e.g. GitHub). See the Nature Portfolio guidelines for submitting code & software for further information.

## Data

Policy information about availability of data

All manuscripts must include a data availability statement. This statement should provide the following information, where applicable:

- Accession codes, unique identifiers, or web links for publicly available datasets
- A description of any restrictions on data availability
- For clinical datasets or third party data, please ensure that the statement adheres to our policy

For the human association studies we have used data from UK Biobank. Access to the UKB Resource is available by application (http://www.ukbiobank.ac.uk/). Access to data from the UK Biobank can be obtained at https://www.ukbiobank.ac.uk/enable-your-research. The UK Biobank resource is available to bona fide researchers for health-related research in the public interest. All researchers who wish to access the research resource must register with UK Biobank by completing the registration form in the Access Management System. Registrations are according to UK Biobank reviewed within 10 working days of submission (https://www.ukbiobank.ac.uk/enable-your-research/register). After registration is approved, applications on access to the UK Biobank research resource can be submitted. Research applications to access the data are performed via the Access Management System (https://bbams.ndph.ox.ac.uk/ams/).

Datasets used for human genetic association studies have been published by Morris et al., Nature Genetics 2019
GWAS summary statistics are available at the GEFOS website http://www.gefos.org
Associations with eQTLs for the three SNPs at the RSPO3 locus were evaluated using the GTEx Portal https://gtexportal.org/home/
The associations for the cis-pQTLs for RSPO3 were taken from two independent datasets, including rs3734626 from Emilsson et al., Science 2018 and rs2489623 from Sun et al., Nature Genetics 2018.

Single cell RNA sequencing (scRNA seq) data: scRNA seq data have previoulsy been deposited in GEO, accession number GSE136970 (https://www.ncbi.nlm.nih.gov/geo/query/acc.cgi?acc=GSE136970, Matsushita el al 2020) and GSE108892 (https://www.ncbi.nlm.nih.gov/geo/query/acc.cgi?acc=GSE108892, Tikhonova et al, 2019).
Source data for mechanistic studies are provided with this paper.

# Field-specific reporting

Please select the one below that is the best fit for your research. If you are not sure, read the appropriate sections before making your selection.

☒ Life sciences ☐ Behavioural & social sciences ☐ Ecological, evolutionary & environmental sciences

For a reference copy of the document with all sections, see nature.com/documents/nr-reporting-summary-flat.pdf

# Life sciences study design

All studies must disclose on these points even when the disclosure is negative.

| | |
|---|---|
| Sample size | Sample size for mechanistic studies where chosen based on previous studies from our group (Movérare-Skrtic et al., Nature Medicine 2014), and the sample sizes used have previously been sufficient to detect differences in the included parameters. For human genetic studies, we used the largest available GWAS. |
| Data exclusions | Data were excluded when technical failures occured in the assays, when there were no double-labeling of the sample for the histomorphometric analyses, when the sample were damaged prior to mechanical testing, or when the mechanical testing failed, by recomendation by the testing technician.<br>Mice were excluded from further analyses if they had been injured by fighting in the cages, or if they were ill after tamoxifen treatment. No other data were purposely excluded, other missing values are due to experimental issues. |
| Replication | The number of replications for in vitro studies are given in the individual figures and as stated in the figures successfully repeated. The number of mice used in the different experiments are indicated in each figure/table. The number of RNA scope micrographs are given in figure 1 and S2. |
| Randomization | The laboratory animals were allocated into groups based on their genotype. The human association studies are population-based studies, so no randomization was performed. |
| Blinding | Animal experiments were run blind (except for the project leader) for the staff performing animal studies. All samples were blinded during analyses. |

# Reporting for specific materials, systems and methods

We require information from authors about some types of materials, experimental systems and methods used in many studies. Here, indicate whether each material, system or method listed is relevant to your study. If you are not sure if a list item applies to your research, read the appropriate section before selecting a response.

## Materials & experimental systems

| n/a | Involved in the study | |
|---|---|---|
| ☐ | ☒ | Antibodies |
| ☒ | ☐ | Eukaryotic cell lines |
| ☒ | ☐ | Palaeontology and archaeology |
| ☐ | ☒ | Animals and other organisms |
| ☐ | ☒ | Human research participants |
| ☒ | ☐ | Clinical data |
| ☒ | ☐ | Dual use research of concern |

## Methods

| n/a | Involved in the study | |
|---|---|---|
| ☒ | ☐ | ChIP-seq |
| ☒ | ☐ | Flow cytometry |
| ☒ | ☐ | MRI-based neuroimaging |

# Antibodies

**Antibodies used**

LRP6 (clone C47E12, Cell Signaling Technology, 3395)
anti-P-LRP6 (Ser1490, Cell Signaling Technology, 2568)
anti-TRAP (clone ZY-9C5, Zymed, MS-1768-R7)

**Validation**

The LRP6 and anti-P-LRP6 antibodies were analyzed by capillary-based electrophoresis and immunodetection using JESS ProteinSimple system and the Compass Software (Protein Simple) as described by the manufacturer.
The LRP6 antibody have previously been published by Mol Biol Cell. 2020 Jun 15;31(13):1425-1436. doi: 10.1091/mbc.E20-02-0114. Epub 2020 Apr 22
The anti-P-LRP6 antibody have previously been published in Nat Commun. 2020 Oct 21;11(1):5321. doi: 10.1038/s41467-020-19173-2.
The anti-TRAP antibody have previously been published in Arthritis Rheum. 2010 May;62(5):1549-56. doi: 10.1002/art.27356.

# Animals and other organisms

Policy information about studies involving animals; ARRIVE guidelines recommended for reporting animal research

**Laboratory animals**

Female and male mice on C57/BL6N background were used in the study.
Mice used:
Rspo3flox/flox: females n=12-14; males, n=14-17. Age: 13-15 weeks at termination.
Runx2-creRspo3flox/flox: females, n=12; males, n=14. Age: 13 weeks at termination.
CAGGCre-ER-Rspo3flox/flox: males, n=10-11. Age: 13 weeks at termination.
Dmp1-creRspo3flox/flox: females, n=16; males, n=14. Age: 15 weeks at termination.

**Wild animals**

No wild animals were used in this study.

**Field-collected samples**

No field-collected samples were used in this study.

**Ethics oversight**

All animal experiments included in this study were approved by the Ethics Committee in Gothenburg, Västra Götaland.

Note that full information on the approval of the study protocol must also be provided in the manuscript.

# Human research participants

Policy information about studies involving human research participants

**Population characteristics**

The UK Biobank is a large prospective cohort study of approximately a half-million adult (ages 40–69 years) participants living in the United Kingdom with genotype, phenotype, and linked health record data, recruited from 22 centers across the United Kingdom in 2006–2010. Our analyses focused on the subset of 438,937 participants of white European descent who had data on the candidate SNPs at the RSPO3 locus, the two fracture outcomes (hip fractures, n=4035, distal forearm fractures, n=7234) and relevant covariates (age, sex, height, weight). Within this population, 46% were male, the mean age at recruitment was 57 years, the mean height was 169 cm, and the mean weight was 78 kg

**Recruitment**

The UK Biobank cohort is a population-based cohort that recruited volunteers. Approximately 9.2 million individuals aged 40–69 years who lived within 25 miles (40 km) of one of 22 assessment centers in England, Wales, and Scotland were invited to enter the cohort, and 5.5% participated in the baseline assessment. UK Biobank participants were more likely to be older, to be female, and to live in less socioeconomically deprived areas than nonparticipants. Compared with the general population, participants were less likely to be obese, to smoke,and to drink alcohol on a daily basis and had fewer self-reported health conditions (PMID: 28641372). Thus, UK Biobank is not representative of the sampling population as there is evidence of a “healthy volunteer” selection bias. Nonetheless, we believe that this selection bias most likely did not substantially impact the identified assocaitions between SNPs at the RSPO3 locus and fracture risk in the present report.

**Ethics oversight**

The UK Biobank has ethical approval from the North West Multi-Centre Research Ethics Committee (16/NW/0274) , and informed consent was obtained from all participants. The present research was approved by the UK Biobank Research and Access Committee (application no. 51784).

Note that full information on the approval of the study protocol must also be provided in the manuscript.
